# Psychometric Properties of the Millon Clinical Multiaxial Inventory–III in an Arabic Clinical Sample Compared With American, Italian, and Dutch Cultures

**DOI:** 10.3389/fpsyg.2021.562619

**Published:** 2021-09-09

**Authors:** Naser Abdulhafeeth Alareqe, Samsilah Roslan, Mohamad Sahari Nordin, Nor Aniza Ahmad, Sahar Mohammed Taresh

**Affiliations:** ^1^Department of Educational Psychology and Counseling, Taiz University, Taiz, Yemen; ^2^Department of Foundations of Education, Faculty of Educational Studies, Universiti Putra Malaysia, Seri Kembangan, Malaysia; ^3^Department of Educational Psychology and Counseling, International Islamic University Malaysia, Selayang, Malaysia; ^4^Department of Kindergarten, Taiz University, Taiz, Yemen

**Keywords:** psychopathology, MCMI-III, advanced factor analysis, internationalization, externalization, psychometrics, tucker's coefficient of congruence

## Abstract

Millon Clinical Multiaxial Inventory MCMI–III is a multidimensional measure of psychopathology with excellent construct validity, test-retest reliability as well as internal consistency. Factor analysis of the MCMI-III has produced mixed results, extracting parsimonious three-factor solutions, or replicating the original four-factor solution in psychiatric samples from Western countries. However, little work has been done on the psychometric properties of the MCMI–III, using non-Western psychiatric samples. Outpatients (*N* = 212) completed the MCMI–III during a semi-structured interview. Eight exploratory factor analysis (EFA) methods were used to explore the underlying structure of MCMI–III. Skewness, kurtosis, and descriptive statistics confirmed that scales of MCMI–III were normally distributed. High-internal consistency was found. The eight EFA methods applied to the 24 clinical scales identified a consensual three-factor solution: factor I (internalizing psychopathology; 18 scales), factor II (externalizing psychopathology; 4 scales) and factor III (psychological disturbance; 2 scales), accounting for a total of 72% of the common variance. Regarding the cross-cultural equivalence of the MCMI–III structure, Tucker's congruence coefficient (Φ) was used and confirmed that internalizing (F1) and externalizing psychopathology (F2) factors obtained in this study are similar to high vs. low psychopathology and emotional constraint factors provided by American study of Haddy et al. (2005) (Φ was 0.86 and 0.97). These two factors are also similar to the general adjustment and antisocial acting out factors provided by the American study of Craig and Bivens (1998) (Φ was 0.82 and 0.96). The first two factors in this study also reflect high similarity with the factor solutions obtained with the Italian and Dutch versions of MCMI-III (Rossi et al., 2007; Pignolo et al., 2017). Despite using a psychiatric sample from a non-Western culture, the two factors identified for this MCMI–III Arabic version were similar to those reported on studies with MCMI–III, using primarily Western samples (Craig and Bivens, 1998; Rossi et al., 2007).

## Introduction

The Millon Clinical Multiaxial Inventory (Millon, 1981) and its revisions, the Millon Clinical Multiaxial Inventory-Second Edition (Millon, 1987), and the MCMI-Third Edition (Millon, 1994; Millon et al., 1997) and Millon Clinical Multiaxial Inventory MCMI–III (Millon et al., 2006) have gained rapid acceptance and popularity as an objective measure of psychopathology (Piotrowski and Lubin, 1990). It is particularly useful for diagnosing personality disorders and dysfunctional relational patterns. Since its introduction in 1977, MCMI has become one of the most widely used and clinical assessments in history, generating nine books (Craig, 2005).

According to Millon et al. (2012), the MCMI has been used in literally hundreds if not thousands of studies to assess personality functioning and to assist in the diagnosis of personality disorders. Only the Rorschach (Exner, 1993) and the Minnesota Multiphasic Personality Inventory−2 (MMPI-2) (Butcher et al., 2006) have produced more research within the past 5 years (Strack, 2002; Craig, 2005). The MCMI, currently, is the second most frequently used personality test in civil (Boccaccini and Brodsky, 2002) and forensic psychology (Craig, 2005).

The evaluation of the clinical assessments quality in a psychopathological research is characterized by sound psychometric properties as reliability (Cronbach's alpha, internal consistency indices, test-retest, and split half) and validity (e.g., construct, content, and convergent). In addition, the clinical assessments are also preferred to be expressive about clinical comprehensive theory and to be able to test alternative theoretical models simultaneously. MCMI–III is characterized by these conditions as well as translated into several languages.

### Clinical Comprehensive Theory

MCMC is based on sound-underlying theory of personality. It was developed to operationalize comprehensive theory of psychopathology of Millon (1983), and it has been revised two times over the past 20 years to keep pace with changes in the theory as well as the development in the Diagnostic and Statistical Manual of Mental Disorders (APA, 1994). Based on his theory, Millon proposed four axes, namely active-passives, pleasure-pain, self-other, and structural pathology as the basic building blocks of normal and abnormal personality. MCMI is designed for assessing the four axes. Finally, Millon et al. (2006) theorized that psychopathology reflected by MCMI–III can be classified under four latent structures or the four-factor model of Millon: the 11 clinical personality disorders, the three severe personality disorders, the seven clinical syndromes, and the three severe clinical syndromes.

#### Psychopathology: the Four-Factor Model of Millon

**Figure 1:**
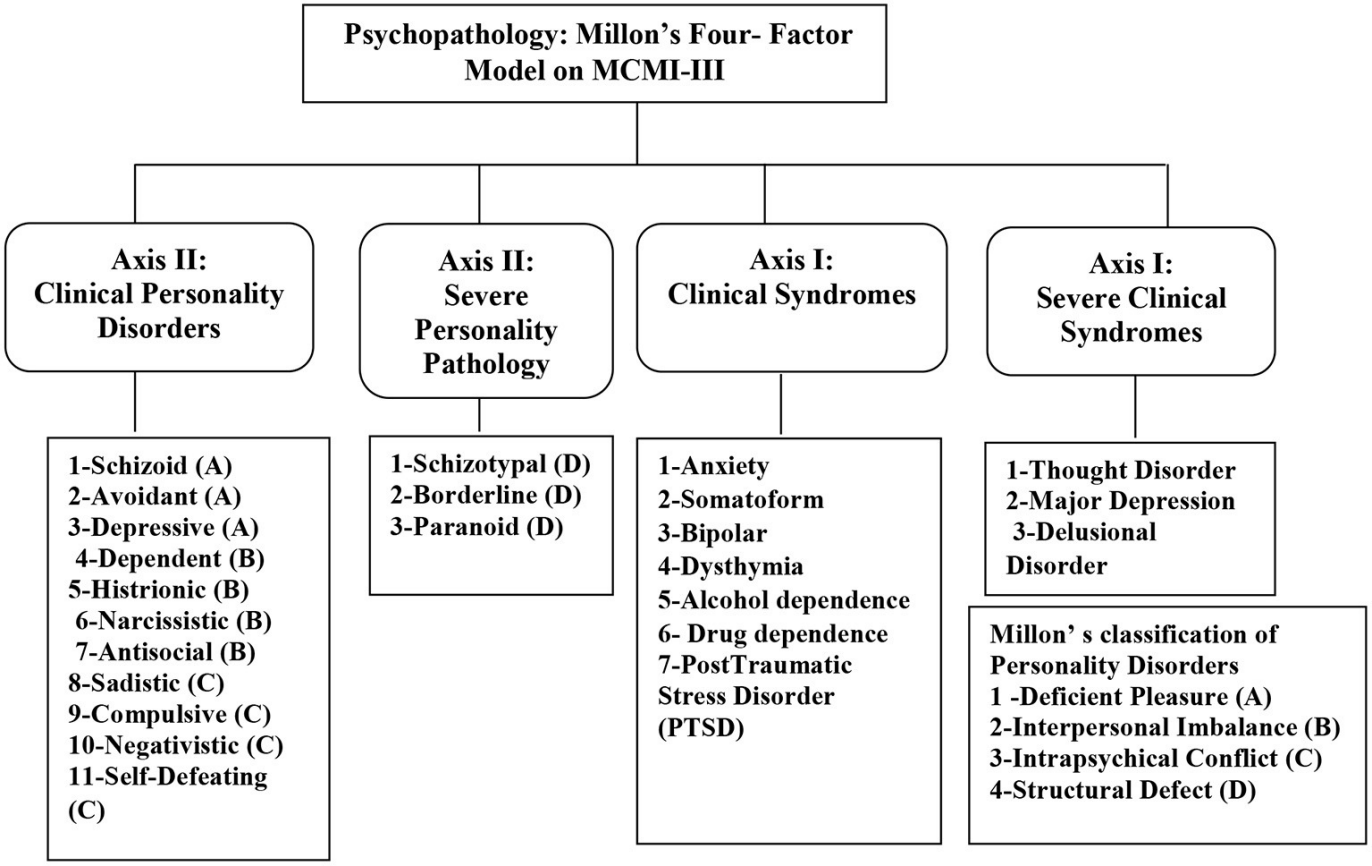
The four-factor model of psychopathology of Millon classified on MCMI-III.

Millon (1997) categorized psychopathology (Figure 1) into two types: clinical syndromes (Axis I) and personality disorders (Axis II). Personality disorders have been broken down in clinical/moderate and severe. Clinical personality disorders (first-factor) include schizoid, avoidant, depressive, dependent, histrionic, narcissistic, antisocial, sadistic, compulsive, negativistic, and self-defeating. Individuals with basic personality disorders may experience mild-to-moderate levels of impairment in their ability to function socially or occupationally, but they may be able to maintain an intimate relationship and continue to work. In contrast, the three severe kinds of personality pathology—(second-factor)-schizotypal, borderline, and paranoid—are usually considerably disabled. Therefore, it is difficult for an individual to score high on these scales to function effectively in social, occupational, or academic areas. As it does with the personality disorders, the MCMI-III breaks clinical syndromes into two categories: clinical/moderate and severe. Clinical syndromes scales (third-factor) include anxiety, somatoform, bipolar, dysthymia (chronic depression), alcohol dependence, drug dependence, and post-traumatic stress disorder (PTSD). Individuals with elevations on the clinical syndrome scales can probably function with mild to moderate impairment. The severe clinical syndromes scales (fourth-factor) include thought disorder, major depression, and delusional disorder. These three scales are designed to reveal more severely debilitating and more complex clinical syndromes. Although Millon assumed theoretically that there are four factors of psychopathology underlying MCMI-III, empirical studies were obtained from two to six factors. Cluster analysis and principal component analysis are used to define the structure of psychopathology, using MCMI-III. Previous studies (e.g., Craig and Bivens, 1998; Rossi et al., 2007) retained three to four-factor solution based on exploratory factor analyses for the MCMI-III and named these factors as internalizing psychopathology/general maladjustment, paranoid behavior/thinking with emotional detachment, and externalizing psychopathology/antisocial acting out.

### Test of Alternative Theoretical Models

The most adequate instruments in psychopathological research can be used as a tool to assess alternative theoretical models. MCMC–III is the only clinical instrument that assesses all personality disorders classified under Axis II and several disorders of Axis I (clinical syndromes) of DSM. The MCMI–III is coordinated with the multiaxial format provided in DSM-IV and is linked to its conceptual terminology and diagnostic criteria, providing diagnostic accuracy (Craig, 2005). Most recently, Rossi and Derksen (2015) indicated that the scales of MCMI–III remain compatible with recently published DSM-5 (APA, 2013).

#### Psychopathology: DSM Multiaxial Model (Axis II and Axis I)

The disorders in the diagnostic and statistical manual of mental disorders (DSM-IV-TR; APA, 2000) are grouped in terms of a multiaxial model. Multiaxial literally means multiple axes; each axis represents different kinds or sources of information. The multiaxial model exists because some meanings are required. The various symptoms and personality characteristics of a given patient can be brought together to portray the picture that reflects the functioning of the whole person. For example, depression in a narcissistic personality is different from depression in a dependent personality. Because narcissists consider themselves superior to everyone else, they usually become depressed when confronted with objective evidence of failure or inadequacy that is too profound to ignore. Their usually puffed-up self-esteem deflates, leaving feelings of depression in its wake. In contrast, dependent personalities seek others who are powerful enough to take care of them—instrumental surrogates who confront a cruel world. Here, depression usually follows the loss of a significant caretaker. The point of the multiaxial model is that each patient is more than the sum of his or her diagnoses: both are depressed, but for very different reasons (Millon et al., 2012).

**Figure 2:**
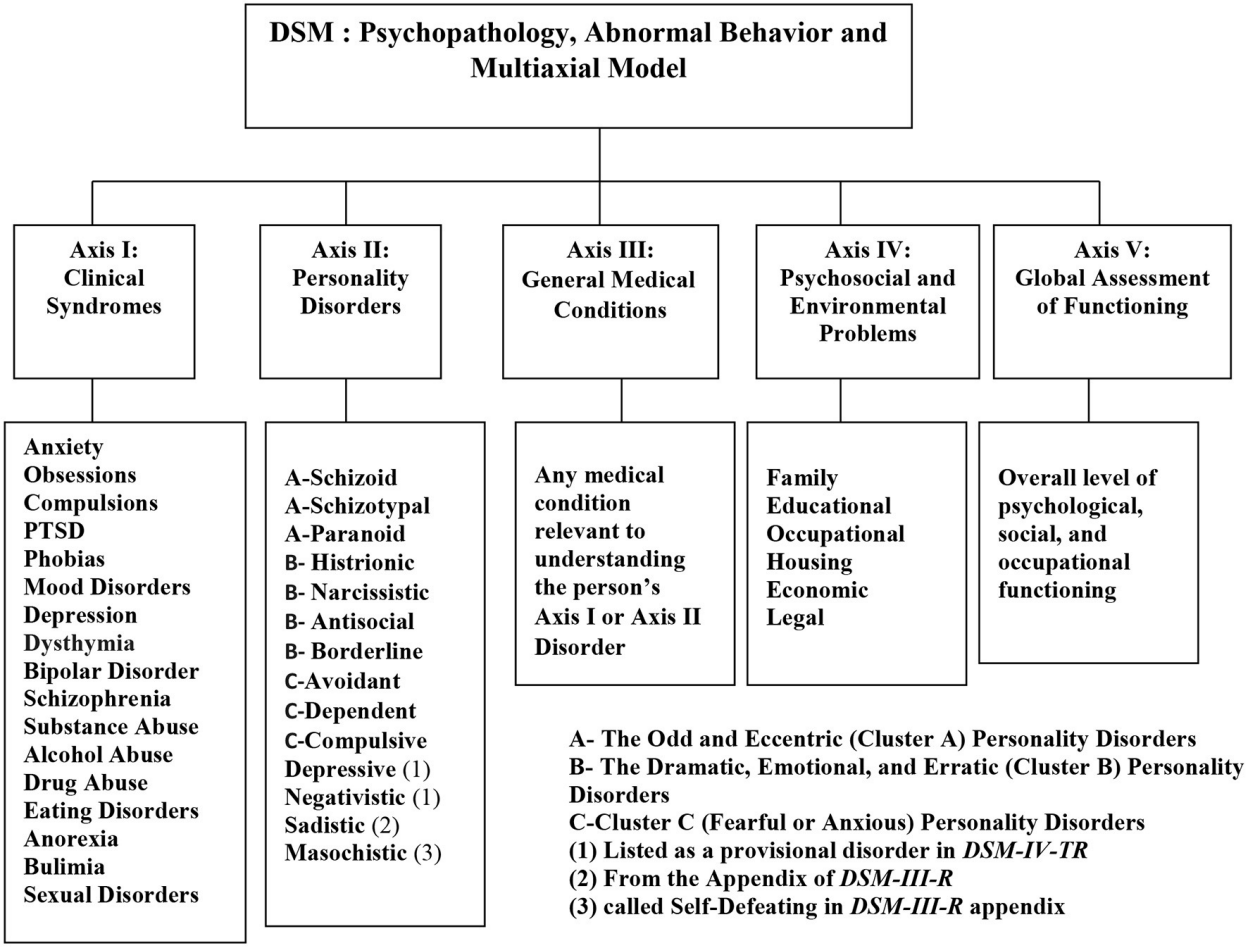
Psychopathology, abnormal behavior, and the multiaxial model.

The multiaxial model is divided into five separated axes (Figure 2); each axis gets at a different source or level of influence in human behavior. Axis I, *clinical syndromes*, consists of the classical mental disorders that have preoccupied clinical psychology and psychiatry for most of the history of these disciplines. Axis I is structured hierarchically; each family of disorders branches into finer distinctions, which compose of actual diagnoses. For example, anxiety disorders include obsessive-compulsive disorder, post-traumatic stress disorder, and generalized anxiety disorder. The mood disorders include depression and bipolar disorder.

Other branches recognize different types of disorders, such as sexual disorders, eating disorders, and substance abuse disorders. Finally, each disorder is broken down into diagnostic criteria, and a list of symptoms must be present for the diagnosis to be accurately given (Millon et al., 2004). Axis II contains 10 personality disorders grouped into three clusters: firstly, paranoid, schizoid, and schizotypal patterns, which are termed as the odd and eccentric personality disorders (Cluster A); secondly, antisocial, borderline, histrionic, and narcissistic patterns, which named as the dramatic, emotional, and erratic personality disorders (Cluster B); and thirdly, avoidant, dependent, and obsessive-compulsive patterns, which called as the fearful or anxious personality disorders (Cluster C) (Segal et al., 2006).

#### Psychopathology: the Internalizing and Externalizing Model for Adults of Krueger

Furthermore, Krueger et al. introduced the objective model for adult psychopathology with two classifications: internalizing and externalizing disorders as more parsimonious than the DSM organizational structure (personality disorders vs. clinical syndromes). Internalizing psychopathology was represented by major depression, dysthymia, generalized anxiety disorder, agoraphobia, social phobia, simple phobia, and obsessive-compulsive disorder. Externalizing psychopathology was indicated by conduct disorder, marijuana dependence, and alcohol dependence (Krueger et al., 1998, 2001, 2016; Krueger, 1999; Kupfer et al., 2008). MCMI–III can be used comprehensively to assess the internalizing and externalizing disorders (Rossi et al., 2007; Rossi and Derksen, 2015).

### Construct Equivalence

Construct equivalence is the foundation of any cross-cultural assessment that intends to produce comparative data. It means that the assessment of the construct provides the same meaning and value for the target participants from different cultures. A shared understanding of the construct gives the participants accurate test conditions and test administrators, which is the best tool to make cross-cultural comparisons (Van de Vijver and Tanzer, 2004). CE focuses on the similarity of concepts and their interpretation across cultures. Although researchers use different labels for this interpretative equivalence, all of them refer to the same issue. CE deals with the theoretical validity of the concepts measured by the survey questions and the item batteries and thus is interpretative equivalence (Davidov et al., 2014). CE is a prerequisite for meaningful cross-national analyses and comparisons due to the fact that the same interpretation of theoretical concepts cannot be made for respondents from different political, social, and cultural contexts (Davidov et al., 2014).

CE can be tested in several ways; for example, by country-specific expert judgments, focus groups, cognitive interviews, statistical tests of item batteries, and open-ended questions in surveys to identify the associations with respective relevant terms (Davidov et al., 2014).

MCMI received attention from researchers from different cultures. MCMI construct equivalence across different cultures was provided by some techniques; for instance, comparability of MCMI-III was presented between two nations: Danish and Belgian, using multiple-group CFA (MG-CFA) (Rossi et al., 2010). A four-factor framework for personality disorders obtained results with partially invariant factor loadings.

Study conducted by Pignolo et al. (2017) aimed to explore the factor structure of the Italian version of the MCMI-III. By comparing the obtained results of the four-factor model with the Dutch and American factor structure, typicality and congruence between the Italian version and these two different cultures were found.

Using principal components analysis and congruence analysis, the Millon Clinical Multiaxial Inventory was applied by Egger and his team (Egger et al., 2003) to 263 Dutch inpatient substance abusers with multiple psychiatric diagnoses, and the results compared with those of 306 North-American substance abusers tested by Ward (Ward, 1995). Degree of similarities between two cultures on MCMI was found based on Tucker's congruence coefficient.

### Factor Analytic Research on MCMI–III

As indicated, there are a huge number of publications about MCMI. Subsequently, this short review reports the studies that used MCMI–III in clinical sample analyzed by factor analysis. We can emphasize the number of factor analytic studies on the Millon Clinical Multiaxial Inventory MCMI–III (Millon et al., 2006) has shown the mixed results, extracting from three to four factors with ≥60% as a proportion of variance explained and ≥0.60 as magnitudes of factor loading.

Sloore et al. (Rossi et al., 2010) have analyzed the factor structure of MCMI–III after translating the MCMI–III into Dutch, using a principal component analysis (with varimax rotation). The MCMI–III was administered to a group of 656 inpatients, outpatients, and prisoners. Four-factor solution was obtained, and 76.37% of the resulting variance was explained. Magnitudes of factor loading were more than 0.60.

Factor 1 seems to point in the direction of a passive and dependent attitude, including depressive, dependent, and masochistic. Factor 2 could be indicative of problematic impulse control, including antisocial, sadistic, compulsive, and borderline. Factor 3 is a component of suspicion and skepticism, including passive-aggressive, schizotypal, and paranoid. Factor 4 seems to be the classical introversion-extraversion dimension: schizoid, avoidant, histrionic, and narcissistic. Results of separate factor analyses on the clinical and forensic populations produced very similar factors, although the range order of the factors is different (Rossi et al., 2010).

Craig and Bivens (1998) have concluded that the MCMI–III scales tap three underlying dimensions from 444 African American male inpatients on a substance-abuse treatment program. Applying principal components analysis with varimax rotation, authors retained a three-factor solution for the MCMI–III, which accounted for 64.5% of the variance in scale scores. Factor 1 had an eigenvalue of 11.57 and accounted for 48.2% of the variance in scores. This largest factor was a bipolar factor, which labeled general maladjustment. The positive pole of this factor was defined by high loadings on the depressive personality, major depression, dysthymic, and avoidant scales, and had significant loading on the somatoform, schizoid personality, dependent personality, borderline, schizotypal, and anxiety scales. Its negative pole is defined by strong negative loading on the histrionic, narcissistic, and compulsive scales.

Factor 2—paranoid behavior/thinking with emotional detachment—had an eigenvalue of 2.24 and accounted for 9.5% of the variance in subscale scores. This factor loaded most heavily on the delusional disorder, paranoid, and schizotypal scales; it also loaded significantly on bipolar: mania, thought disorder, passive-aggressive, anxiety, post-traumatic stress, borderline, avoidant, and dependent.

Factor 3 had an eigenvalue of 1.63 and accounted for 6.8% of the variance in scores. This factor was a bipolar factor, which labeled antisocial acting out. Its positive pole loaded most heavily on the antisocial, drug dependence, alcohol dependence, aggressive/sadistic, and the bipolar: manic scales. Its negative pole was defined by the compulsive scale.

Haddy et al. (2005) have concluded that the MCMI–III personality disorder scales tap three underlying dimensions. They applied principal components analysis with varimax rotation to base rate (BR) scores, and to non-overlapping scores. Using the BR scores, three factors were retained. The first factor was labeled low versus high emotional constraint, the second was social detachment/introversion versus extraversion, and the third was high versus low neuroticism. Of more importance are the results using non-overlapping scores, since these can be compared with Dyce et al. (1997).

Three factors were retained. The first factor—social detachment/introversion vs. extraversion—was most strongly defined by positive weights for the avoidant, depressive, schizotypal, masochistic, borderline, and paranoid scales. The second factor was called “hostile dominance” and had positive loading on the narcissistic, histrionic, sadistic, paranoid, and antisocial scales. The third factor—low vs. high emotional constraint—had the antisocial scale on one end, and the compulsive scale on the other end.

Moreover, Grossman (2004) has employed an alpha factoring technique with oblique rotation to factor-analyze the items of the individual PD scales of MCMI–III, using the normative sample of the test. Grossman (2004) was able to recover two or three subscale elements for each personality prototype. The prototype subscales were later refined into facet subscales, using rational and empirical criteria. Significantly, the prototype subscales were found to measure major structural and functional elements of the personalities as outlined by Millon (1983).

More significantly, Rossi et al. (2007) have analyzed the factor structure of the Dutch-language version of the MCMI–III (Millon et al., 2006), using exploratory factor analysis with direct oblimin rotation. The authors retained four factors. The extracted factors explained 75.97% of the total variance (Factor 1 = 52.61%; Factor 2 = 14.23%; Factor 3 = 5.09%; and Factor 4 = 4.04%). Factor 1 contained the following scales: depressive, dependent, negativistic, masochistic, schizotypal, anxiety, somatoform, bipolar: manic, dysthymia, post-traumatic stress, thought disorder, and major depression. Their factor loading had a fair magnitude. Similar to the previous study (Craig and Bivens, 1998), Rossi et al. (2007) labeled this factor as general maladjustment. Also, the authors indicated that a plausible interpretation is that General Maladjustment Factor points to an underlying dimension of internalizing disorders.

The antisocial, aggressive, alcohol dependence, and drug dependence scales had fair-sized or higher positive loading on Factor 2, while the compulsive scale had negative loading on Factor 2. Analogous with the previous study (Craig and Bivens, 1998), the authors termed this factor as aggression/social deviance. The paranoid and delusional disorder scales had higher positive loading on Factor 3. Similar to the previous study (Craig and Bivens, 1998), Rossi et al. (2007) named this factor as paranoid/delusional thinking. Finally, the histrionic and narcissistic scales had higher positive loading on Factor 4, whereas the schizoid and avoidant scales had negative loading. Comparable with the previous study (Craig and Bivens, 1998), the authors labeled this factor as emotional instability/detachment.

In brief, a number of the studies used exploratory factor analysis on MCMI–III, using a clinical sample from different countries in Western culture, resulting in three to four factors. However, it is presently unclear about the psychometric properties of the MCMI–III, using a non-Western psychiatric sample. Supporting this idea, Rossi and Derksen (2015) reviewed a huge number of publications on MCMI. They found shortcomings for MCMI–III studies, using a clinical sample from non-Western countries. For example, Nasiri et al. (2013) used the Persian version of the MCMI–III with 50 Iranian women characterized by a mental health problem. However, no parameters of psychometrics properties were available.

### Aims of the Study

As previously mentioned, several studies on the factor structure of MCMI-III were conducted within the context of the United States and European countries (Rossi and Derksen, 2015). However, there is a scarcity of research in MCMI-III outside of Western countries, particularly about its psychometric properties. Moreover, some previous studies revealed that the scores of some MCMI-III scales do not achieve univariate and multivariate normal distribution assumptions. Thus, the current study aims to test the normal distributions of the scales of the MCMI–III to evaluate their internal consistency and to explore the factor structure of MCMI–III, using a non-Western psychiatric/clinical sample. It also aims to compare this factor structure with those obtained in studies conducted within the western context.

## Methods

### Millon Clinical Multiaxial Inventory

MCMI–III (Millon, 1994) is self-report personality and diagnostic inventory, which is designed to assess 14 personality disorders and 10 clinical syndromes. It has four modifying indices. MCMI–III is a 175-item, true-false, self-administered questionnaire that assesses Axis I (10 clinical syndromes) and Axis II (14 personality disorders) based on the *Diagnostic and Statistical Manual of Mental Disorders*) (APA, 2000).

The MCMI–III has adequate internal consistency (alphas ranging from 0.66, for the compulsive scale, to 0.90 for the major depression scale) (Millon et al., 2006). Test-retest reliability of the MCMI–III scales was obtained by readministering the scale before 5 to 14 days after the initial administration to 0.87 subjects. The reliability ranged from 0.84 for the anxiety scale to 0.96 for the somatoform scale. The median stability coefficient was 0.91, which suggests that the MCMI–III results are highly stable over a short period of time (Millon et al., 2006).

The validity of MCMI–III has been established by using a norming sample of 998 patients. The MCMI–III demonstrated strong correlations between clinician ratings and various MCMI–III scales, especially for syndromes that are easily identified and can be detected with minimal diagnostic interviewing. Significant correlations were also obtained between the MCMI–III scales and various instruments (e.g., Minnesota Multiphasic Personality Inventory-II, Symptom Distress Checklist-90-R, Beck Depression Inventory, General Behavioral Inventory, etc.) that purport to measure similar constructs (Millon et al., 2006).

Finally, there was a strong degree of concordance between MCMI–III items and specific diagnostic criteria for the Diagnostic and Statistical Manual of Mental Disorders (APA, 2000; Millon et al., 2006). The data showed that 84% (105) of the 125 relevant diagnostic criteria statements in DSM (myth of mental disorders for psychiatrists and clinical psychologists) have a direct corresponding item on the MCMI–III (Hsu, 2002, 2005; Millon et al., 2006). Moreover, there were no differences between MCMI–III subscales and DSM–IV-TR Diagnosis (Millon et al., 2006) and DSM-V (Rossi and Derksen, 2015).

#### Content Validity of MCMI-III

The primary measurement scales of MCMI-III can be described as the multifaceted psychological constructs adhering to principles of the evolutionary theory of Millon (1990) that demonstrate the construct and content validity consonant both with the overarching theory and the DSM-IV-TR diagnostic categories. As stated earlier, the scales of MCMI–III remain compatible with the recently published DSM (APA, 2013).

In the second edition of the MCMI-III manual, Millon et al. (1997) described the process leading to the development of the various versions of the MCMI. Suffice it to say that the original MCMI was based on a clearly outlined model of personality and psychopathology. The research team was selected from a pool of over 3,500 items to find sets matching each clinical construct and then evaluated by a panel of eight mental health professionals. The combination of a clearly stated theory and careful procedures with empirical checks is strongly suggestive of good content validity. One of the critics for MCMI-III is to contain overlapping items among mental disorders; however, the clinical reality of comorbidity among psychopathological disorders cannot be ignored; Millon tried to include this reality in his instruments by using overlapping items (Rossi and Derksen, 2015).

### Procedure

The MCMI–III was administered by the first author and clinical psychiatrists during the psycho-diagnostic evaluation session for 4 months. During the psych-diagnostic evaluation sessions, counseling techniques and clinical interventions, such as active listening, empathy, rephrase, and feedback, were recruited in high commitments. Following the instruction of the use of MCMI–III in its Manual (Millon et al., 2006), profiles are considered valid if the total number of omitted or invalid responses (e.g., both a “yes” response and a “no” response to a single item) was <12, if the validity index was <2, and if the raw score on Scale X (Disclosure) was within the range 34–178 (Millon et al., 2006). These scales represented the inclusion/exclusion criteria for acceptance/removing the response of the patient on MCMI-III. Raw scores are more appropriate than base rate (BR) scores at using statistical analyses, which require interval measurement of the data, such as factor analysis (Hsu, 2005; Millon et al., 2006).

As an Arabic version of the MCMI–III was not available when the current study was carried out, the MCMI–III was translated to Arabian by the author of this study and two psychology professors with adequate bilingual languages under the supervision of the English Center of International Islamic University Malaysia. First and back translations were conducted, and the discrepancies between two versions were solved.

### Statistical Analysis

#### Descriptive Statistics

Skewness and kurtosis indices are statistics used to describe the symmetry and shape of the distribution. Normal distribution has skewness and kurtosis coefficients with value of 0.0. So, when the magnitude of those coefficients is small (<1), they suggest a normal distribution (Tabachnick et al., 2007). Furthermore, Gravetter et al. (2016) stated the measures of central tendency: the mean, median, and mode can be used as indicators for a typically symmetrical distribution. The mean and median fall at the same point and are equal if the data had the typically symmetrical distribution and the value of the mode is not different from the mean and median.

Cronbach's alpha is a method for estimating the reliability of a measure through internal consistency. Generally, Cronbach's alpha of 0.70 is the acceptable standard value (Trochim, 2006).

#### Factor Analysis

Raw data were factors analyzed in IBM SPSS Statistics 22, using the four extraction methods of factor analysis: principal component analysis (PCA), principal axis factoring (PAF), alpha factoring (AF), and unweighted least squares (ULS) cross the orthogonal and oblique rotation procedures.

Multicollinearity and singularity may cause both logical and statistical problems in factor analysis (Tabachnick et al., 2007). The correlation matrix of the measure was free from coefficients equals or above 0.90, meaning that multicollinearity and singularity assumptions were absent and not a threat.

Assumptions of factor analysis are briefly discussed. Initially, Kaiser–Meyer–OIkin (KMO) measure of sampling adequacy indicated whether or not enough factors were predicted by each component. The Kaiser–Meyer–OIkin (KMO) measure should be more than 0.70. Its value of more than 0.90 indicates to a superb level (Leech et al., 2015). Moreover, Bartlett's Test of Sphericity was significant (e.g., significance value of <0.05) (*p* < 0.001), demonstrating correlations among measure variables were adequate to provide a reasonable basis for factor analysis. Diagonal elements of the anti-image correlation should be more than 0.50 (Field, 2013).

Finally, the communalities displayed the relationship between the variable and all other variables due to the common factors. The communality for each item corresponds to the sum of the squared multiple correlations (SMC) across the row of the factor matrix (Field, 2013). Initial communalities of principal component analysis (PCA) always equalized to 1., while initial communalities of principal axis factoring (PAF), alpha factoring (AF), and unweighted least squares (ULS) were <1. Final communalities should be higher than 0.50, and, when a variable shows a communality <0.50, it should be deleted if the magnitude of its highest factor loading was <0.33 (Thompson, 2004).

To determine the adequately extracted dimension/factor-solution, which is directly relevant to factor structure and good validity of the measure, several criteria are used as follows: (1) Consistency of results across factor analysis extraction and rotations methods, (2) Kaiser's eigenvalue >1, (3) adequate proportion of total variance explained (>0.60) (Hair et al., 2009), (4) Cattell's Scree Plot for eigenvalues, (5) Number of factors per component (2 and more) (Stevens, 2012), (6) magnitude of factor loading (>0.55), and (7) *a priori* criteria related to theoretical foundation.

Excellent directions were followed in this study for applying the Factor Analysis. During the next few runs, the researchers experimented with different numbers of factors, different extraction techniques, and both orthogonal and oblique rotations. In brief, output of the different methods of extraction (PCA, PA, AF, and ULS) gives similar results if the data set is good. Also, the different methods of rotation (Varimax orthogonal and oblique) give similar results if the pattern of correlations in the data is fairly clear (Goldberg and Digman, 1994; Tabachnick et al., 2007). Besides, multi-technique approach of factor analysis in the context of MCMI has already been used with MCMI by Rossi et al. (2007).

Although Millon hypothesized that there are four factors of psychopathology underlying the MCMI-III, DSM classified the psychopathology into Axis I and Axis II. Past studies explored several models of psychopathology under MCMI-III. Results of confirmatory strategies for testing these models were problematic in terms of inadmissible solution, failing to converge to a solution. In this regard, Pignolo et al. (2017) stated that objective suggestion for testing MCMI-III is an exploratory strategy.

#### Tucker's Congruence Coefficient for Factorial Similarity

It was initially suggested by Burt and became popular as Tucker's congruence coefficient (Φ) (Tucker, 1951) or Burt–Tucker's congruence coefficients. The congruence coefficient is a meaningful index of factor similarity. It is the cosine of the angle between the two vectors and can be interpreted as a standardized measure of proportionality of elements in both vectors. It is evaluated as:

$$\begin{array}{l} \Phi \left(x,y\right)=\frac{\sum x_{i}y_{i}}{\sqrt{\sum_{}x_{i}^{2}\sum y_{i}^{2}}} \end{array}$$

where Xi and Y_i_ are the loadings of variable *i* on factors x and y, respectively, *I* = 1, …, n. Usually, the two vectors are columns of a pattern matrix obtained by factor analysis. However, they could also be columns of a structure matrix (Lorenzo-Seva and Ten Berge, 2006).

Factor analysis studies, which involve the same variables, applied to subjects from different populations or in different experimental conditions, often require factor interpretations to be compared. Tucker's congruence coefficient (Tucker, 1951) has the objective function for interpreting the compared factors cross groups or cultures. Multigroup confirmatory factor analysis (CFA) is the best way of testing hypotheses of the equivalence of factors. However, when the sample size is large, any hypothesis of equal factors will systematically be rejected. MCFA fails to get solution in a complex instrument with many scales. Subsequently, exploratory factor analysis (EFA) might be more appropriate as a basis for factor comparisons than the CFA approach in most applications, especially in large multidimensional solutions that do not approach very simple structures. In brief, the factor analysis with Tucker's congruence coefficient is the most popular tool for such comparisons.

Lorenzo-Seva and Ten Berge (2006) suggest that a value of Tucker's congruence coefficient (Φ) in the range (0.85–0.94) corresponds to a fair similarity, while a value higher than 0.95 implies that the two factors or components compared can be considered equal. The guidelines given by Tucker: 0.98 to 1.00 = excellent; 0.92 to 0.98 = good; 0.82 to 0.92 = borderline; 0.68 to 0.82 = poor; and below 0.68 = terrible. According to Ten Berge (1986), If Tucker's Φ exceeds 0.85, component loadings similarity can be concluded (cited in Egger et al., 2003).

## Results

### Subjects

The subjects of this study consisted of 212 Yemenis Arab outpatients in Taiz Hospital and private psychiatry clinics in the Yemen Arab country. Average age of the clinical sample was 30.81, with a standard deviation of 7.322, and ranged from 20 to 50. The majority of the participants were married (56.63%), while 34% were single and 9.4% were divorced. Approximately, 86.3% of the sample were male. About 28.3% of the sample have basic education (writing and reading skills), 23.1% have primary education, 35.4% hold secondary a school certificate, and 13.2% are degree holders (Table 1).

**Table 1:** Sociodemographic variables of sample.

**Variables**	**Frequency**	**Percent**
Gender		
Male	183	86.3
Female	29	13.7
Total	212	100.0
Marital status		
Single	72	34.0
Married	120	56.6
Divorced	20	9.4
Total	212	100.0
Study levels		
Basic education	60	28.3
Primary school	49	23.1
Secondary school	75	35.4
University	28	13.2
Total	212	100.0

### Normal Distribution of MCMI–III

The skewness and kurtosis indices indicated that values of 24 scales of MCMI–III in a clinical sample were <1, suggesting normal and symmetrical distributions (Table 2). The values of mean and median of each factor of MCMI–III were identical, with no big differences with mode, indicating the distribution of 24 factors of MCMI–III was drawn normally.

**Table 2:** Skewness and kurtosis, mean, median, and mode, standard deviation, and reliability of scales of MCMI–III.

**MCMI–III-Scale**	**Items**	**Skewness**	**Kurtosis**	**Mean**	**Median**	**Mode**	**Std. deviation**	**Cronbach's alpha**	
I (Schizoid)	16	−0.33	−0.33	11.50	12.00	13.00	4.22	0.804	0.830
2A (Avoidant)	16	−0.43	−0.23	12.72	13.00	15.00	4.43	0.797	
2B (Depressive)	15	−0.25	−0.48	11.98	12.50	14.00	5.30	0.792	
3 (Dependent)	16	−0.23	−0.50	13.64	14.00	16.00	4.37	0.796	
4 (Histrionic)	17	−0.26	−0.10	13.49	14.00	14.00	3.26	0.866	
5 (Narcissistic)	24	−0.21	−0.18	12.24	12.65	13.32	2.25	0.841	
6A (Antisocial)	17	0.03	−0.61	10.97	11.00	11.00	4.86	0.801	
6B (Sadistic)	20	−0.42	0.00	15.24	16.00	15.00	4.87	0.785	
7 (Compulsive)	17	−0.27	−0.40	15.87	16.00	14.00	3.66	0.873	
8A (Negativistic)	16	−0.40	0.16	15.15	15.00	13.00	4.77	0.795	
8B (Masochistic)	15	−0.16	−0.76	10.09	11.00	11.00	4.94	0.784	
S (Schizotypal)	16	−0.35	−0.47	13.21	14.00	17.00	5.68	0.709	0.842
C (Borderline)	16	−0.20	−0.77	12.52	13.00	18.00	5.21	0.785	
P (Paranoid)	17	−0.58	0.04	16.70	17.00	17.00	4.85	0.832	
A (Anxiety)	14	−0.40	−0.42	11.18	11.00	14.00	4.71	0.892	0.913
H (Somatoform)	12	−0.37	−0.54	8.86	10.00	10.00	4.14	0.899	
N (Bipolar):Manic	13	−0.02	−0.48	9.25	9.00	10.00	3.95	0.898	
D (Dysthymia)	14	−0.22	−0.68	9.39	10.00	10.00	4.74	0.893	
B (Alcohol dependence)	15	0.30	−0.61	8.62	8.00	5.00	3.84	0.903	
T (Drug dependence)	14	0.51	−0.85	7.09	6.00	3.00	4.68	0.917	
R (Post-Traumatic stress)	16	−0.22	−0.87	10.22	11.00	11.00	5.20	0.894	
SS (Thought disorder)	17	−0.32	−0.72	11.78	12.00	12.00	5.20	0.703	0.842
CC (Major depression)	17	−0.23	−0.54	11.03	12.00	12.00	5.21	0.724	
PP (Delusional disorder)	13	−0.11	−0.34	9.53	9.50	11.00	3.32	0.864	

### Internal Consistency of MCMI–III

Overall reliability of the 11 clinical personality disorders was 0.830, ranging from 0.78 for masochistic personality disorder, to 0.87 for both histrionic personality and compulsive personality disorders (Table 2). In terms of the three severe personality disorders, overall reliability was 0.842, varying from 0.71 for schizotypal personality disorder to 0.83 for paranoid personality disorder.

Regarding the three clinical syndrome, overall reliability was 0.913, spreading from 0.89 for anxiety disorder, dysthymia disorder, and post-traumatic stress disorder, to 0.92 for drug dependence.

With reference to the seven severe clinical syndrome, overall reliability was 0.842, extending from 0.70 for thought disorder, to 0.86 for delusional disorder.

In general, Cronbach's alpha for the 24 factors cross four domains of psychopathology classification was within acceptable standard value (0.70), ranging from 0.70 to 0.90, which indicated that the factors of MCMI–III in a clinical sample had reasonable internal consistency reliability.

Moreover, the internal consistency of test scales refers to how well the items measure the same latent construct (Nunnally, 1978). High internal consistency (e.g., coefficient α ≥ 0.80) is expected to measure the stable of personality characteristics to reflect the cohesiveness of the underlying traits. Lower levels of internal consistency (e.g., coefficient α ≥ 0.70) are acceptable for the research instruments and the measures of less stable traits in abnormal populations (Strack and Millon, 2007). Four latent constructs (the 11 clinical personality disorders, the three severe personality disorders, the seven clinical syndromes, and the three severe clinical syndromes) were cohesively reflected by a set of their corresponding scales. Twenty-four scales of MCMI-III (e.g., coefficient α ≥ 0.80) obtained high internal consistency, reflecting the cohesiveness of the underlying trait of psychopathology.

### Correlation of MCMI–III

Correlation matrix of a raw score of MCMI–III presented how each of the 24 scales was associated with each of the other 23 scores. The coefficients of correlations ranged from −0.01 for both post-traumatic stress scale and thought disorder, and narcissistic personality disorder to 0.81 between thought disorder and schizotypal personality disorder (Table 3). Although most disorders were moderately interrelated, evidence of multicollinearity ≥(0.90) was not available among variables in correlation matrix of the MCMI–III, meaning that multicollinearity and singularity assumptions were absent and not a threat (Tabachnick et al., 2007).

**Table 3:** Correlation matrix of MCMI–III.

**MCMI–III-Scale**	**I**	**2A**	**2B**	**3**	**4**	**5**	**6A**	**6B**	**7**	**8A**	**8B**	**S**
I (Schizoid)	1.00											
2A (Avoidant)	0.61	1.00										
2B (Depressive)	0.65	0.70	1.00									
3 (Dependent)	0.56	0.64	0.64	1.00								
4 (Histrionic)	−0.51	−0.43	−0.34	−0.22	1.00							
5 (Narcissistic)	−0.07	−0.12	−0.04	−0.03	0.35	1.00						
6A (Antisocial)	0.58	0.44	0.49	0.51	−0.09	0.15	1.00					
6B (Sadistic)	0.54	0.61	0.62	0.61	−0.14	0.17	0.72	1.00				
7 (Compulsive)	−0.33	−0.13	−0.29	−0.14	0.26	0.12	−0.52	−0.27	1.00			
8A (Negativistic)	0.56	0.62	0.61	0.55	−0.23	0.06	0.56	0.74	−0.27	1.00		
8B (Masochistic)	0.68	0.69	0.76	0.67	−0.24	−0.03	0.67	0.67	−0.32	0.61	1.00	
S (Schizotypal)	0.62	0.73	0.75	0.59	−0.29	0.01	0.55	0.64	−0.25	0.63	0.70	1.00
C (Borderline)	0.65	0.65	0.75	0.67	−0.22	0.05	0.68	0.71	−0.34	0.70	0.77	0.71
P (Paranoid)	0.45	0.68	0.58	0.50	−0.14	0.15	0.34	0.62	0.06	0.64	0.56	0.65
A (Anxiety)	0.52	0.68	0.72	0.60	−0.23	0.06	0.49	0.61	−0.18	0.61	0.70	0.71
H (Somatoform)	0.59	0.65	0.68	0.52	−0.30	−0.06	0.47	0.59	−0.19	0.56	0.64	0.62
N (Bipolar):Manic	0.57	0.60	0.66	0.56	−0.20	0.06	0.62	0.69	−0.24	0.66	0.69	0.67
D (Dysthymia)	0.64	0.67	0.77	0.59	−0.34	−0.06	0.54	0.63	−0.29	0.66	0.72	0.75
B (Alcohol dependence)	0.58	0.47	0.53	0.48	−0.16	0.08	0.80	0.61	−0.47	0.48	0.67	0.54
T (Drug dependence)	0.51	0.40	0.41	0.41	−0.06	0.09	0.84	0.56	−0.44	0.38	0.62	0.44
R (Post-Traumatic stress)	0.56	0.68	0.78	0.61	−0.24	−0.01	0.47	0.59	−0.18	0.54	0.69	0.73
SS (Thought disorder)	0.67	0.70	0.79	0.64	−0.29	−0.01	0.59	0.65	−0.32	0.67	0.74	0.81
CC (Major depression)	0.64	0.70	0.75	0.57	−0.28	−0.08	0.52	0.61	−0.25	0.62	0.69	0.68
PP (Delusional disorder)	0.49	0.56	0.59	0.54	−0.13	0.27	0.49	0.59	−0.10	0.51	0.55	0.66
**MCMI**–**III-scale**	**C**	**P**	**A**	**H**	**N**	**D**	**B**	**T**	**R**	**SS**	**CC**	**PP**
C (Borderline)	1.00											
P (Paranoid)	0.55	1.00										
A (Anxiety)	0.77	0.63	1.00									
H (Somatoform)	0.68	0.53	0.69	1.00								
N (Bipolar):Manic	0.75	0.56	0.70	0.62	1.00							
D (Dysthymia)	0.76	0.59	0.74	0.75	0.65	1.00						
B (Alcohol dependence)	0.70	0.33	0.54	0.49	0.61	0.57	1.00					
T (Drug dependence)	0.59	0.26	0.42	0.41	0.48	0.45	0.77	1.00				
R (Post-Traumatic stress)	0.75	0.59	0.81	0.69	0.66	0.70	0.55	0.44	1.00			
SS (Thought disorder)	0.82	0.61	0.78	0.69	0.69	0.83	0.61	0.50	0.77	1.00		
CC (Major depression)	0.76	0.58	0.72	0.83	0.66	0.84	0.60	0.44	0.76	0.76	1.00	
PP (Delusional disorder)	0.61	0.70	0.62	0.54	0.60	0.61	0.49	0.43	0.59	0.63	0.60	1.00

### Eight Methods From Factor Analysis

The Kaiser–Meyer–OIkin measure was 0.952, a superb value indicating that the present data are appropriate for factor analysis. Bartlett's test of sphericity was significant (p < 0.001), demonstrating non-null correlations among MCMI–III scales, a requirement for factor analysis. The diagonal elements of the anti-image correlation matrix were above the acceptable standard value (0.50) for each MCMI–III scale.”

The eigenvalues of the four extraction methods of factor analysis: principal component analysis (PCA), principal axis factoring (PAF), alpha factoring (AF), and unweighted least squares (ULS) produced an initial three-factor solution, which accounted for by about 72.11% of the total variance. Their scree-plots (Figures unshown here) additionally supported the three-factor solution.

The communities across the four extraction methods of factor analysis: principal component analysis (PCA), principal axis factoring (PAF), alpha factoring (AF), and unweighted least squares (ULS) were high for all scales, indicating that the extracted components represented the variables very well. However, three scales (histrionic personality, narcissistic personality, and compulsive personality scales) showed communalities <0.50 by three extraction methods: PAF, AF, and ULS (Table 4). Since the magnitude of their factor loadings was above 0.33 across the eight factor analysis techniques, there is no reason to eliminate them from the analysis.

**Table 4:** Communalities by extraction methods (PCA, PAF, AF, and ULS) for MCMI–III.

**MCMI–III-Scale**	**Principal component analysis (PCA)**		**Principal axis factoring (PAF)**		**Alpha factoring (AF)**	**Unweighted least squares (ULS)**
	**Initial**	**Extraction SMC**	Initial^*^	**Extraction SMC**	**Extraction SMC**	**Extraction SMC**
I (Schizoid)	1.000	0.657	0.690	0.629	0.631	0.629
2A (Avoidant)	1.000	0.727	0.743	0.695	0.716	0.695
2B (Depressive)	1.000	0.776	0.783	0.770	0.764	0.770
3 (Dependent)	1.000	0.555	0.599	0.525	0.525	0.525
4 (Histrionic)	1.000	0.641	0.530	0.449	0.471	0.448
5 (Narcissistic)	1.000	0.647	0.355	0.370	0.366	0.371
6A (Antisocial)	1.000	0.890	0.855	0.932	0.934	0.932
6B (Sadistic)	1.000	0.715	0.752	0.696	0.697	0.696
7 (Compulsive)	1.000	0.657	0.472	0.435	0.440	0.435
8A (Negativistic)	1.000	0.599	0.706	0.575	0.573	0.575
8B (Masochistic)	1.000	0.755	0.772	0.742	0.741	0.742
S (Schizotypal)	1.000	0.736	0.758	0.721	0.718	0.721
C (Borderline)	1.000	0.814	0.821	0.807	0.810	0.807
P (Paranoid)	1.000	0.746	0.735	0.717	0.719	0.717
A (Anxiety)	1.000	0.746	0.778	0.729	0.726	0.729
H (Somatoform)	1.000	0.655	0.732	0.629	0.624	0.629
N (Bipolar):Manic	1.000	0.674	0.680	0.653	0.653	0.653
D (Dysthymia)	1.000	0.781	0.818	0.775	0.769	0.775
B (Alcohol dependence)	1.000	0.791	0.744	0.755	0.759	0.755
T (Drug dependence)	1.000	0.783	0.774	0.731	0.706	0.731
R (Post-Traumatic stress)	1.000	0.728	0.788	0.710	0.705	0.710
SS (Thought disorder)	1.000	0.808	0.830	0.804	0.800	0.804
CC (Major depression)	1.000	0.753	0.845	0.742	0.737	0.742
PP (Delusional disorder)	1.000	0.668	0.671	0.630	0.638	0.630

*SMC, squared multiple correlation*;

* *initial values are the same for AF and ULS*.

The first factor—internalizing psychopathology (eigenvalue = 13.697)—accounted for 57.1% of the variance and had 18 scales with excellent loading, exceeding.55 (Table 5). This factor includes paranoid (P) loadings ≥0.82, anxiety (A) loadings ≥0.82, post-traumatic stress (R) (loadings ≥0.80), depressive (2B) (loadings ≥0.80), thought disorder (SS) loadings ≥0.80, schizotypal (S) loadings ≥0.80, dysthymia (D) loadings ≥0.80, avoidant (2A) loadings ≥0.79, major depression (CC) loadings ≥0.79, borderline (C) (Loadings ≥0.74), somatoform (H) loadings ≥0.73, delusional disorder (PP) loadings ≥0.73, bipolar: manic (N) loadings ≥0.71, masochistic (8B) loadings ≥0.69, negativistic (8A) loadings ≥0.69, dependent (3) loadings ≥0.67, sadistic (6B) loadings ≥0.65, and schizoid (I) scales loadings ≥0.57. The orthogonal and oblique rotation methods of principal component analysis (PCA), principal axis factoring (PAF), alpha factoring (AF), and unweighted least squares (ULS) confirmed the same factors in the same direction (positive pole) with excellent factor loadings of.55 and above.

**Table 5:** Factor loadings for the MCMI-III scales under four extraction and two rotation methods.

**MCMI–III-Scale**	**Varimax**				**Oblimin**			
	**(Rotated factor loadings)**				**(Pattern mattrix)**			
	**PCA**	**PAF**	**AF**	**ULS**	**PCA**	**PAF**	**AF**	**ULS**
**Factor 1: Internalizing psychopathology**								
P (Paranoid)	0.84	0.82	0.83	0.82	0.92	0.92	0.93	0.92
A (Anxiety)	0.84	0.83	0.82	0.83	0.88	0.87	0.87	0.87
R (Post-Traumatic stress)	0.82	0.81	0.80	0.81	0.86	0.85	0.85	0.85
2B (Depressive)	0.82	0.81	0.80	0.81	0.86	0.85	0.84	0.85
SS (Thought disorder)	0.82	0.81	0.80	0.81	0.84	0.83	0.82	0.83
S (Schizotypal)	0.82	0.80	0.80	0.80	0.85	0.83	0.83	0.84
D (Dysthymia)	0.81	0.80	0.80	0.80	0.84	0.83	0.83	0.83
2A (Avoidant)	0.81	0.79	0.80	0.79	0.87	0.85	0.86	0.85
CC (Major depression)	0.81	0.79	0.79	0.79	0.84	0.82	0.82	0.82
C (Borderline)	0.77	0.76	0.76	0.76	0.75	0.74	0.74	0.74
H (Somatoform)	0.76	0.73	0.74	0.73	0.79	0.77	0.77	0.77
PP (Delusional disorder)	0.75	0.73	0.74	0.73	0.79	0.77	0.77	0.77
N (Bipolar):Manic	0.73	0.71	0.71	0.71	0.73	0.71	0.71	0.71
8B (Masochistic)	0.72	0.71	0.71	0.71	0.71	0.69	0.69	0.69
8A (Negativistic)	0.72	0.70	0.69	0.70	0.73	0.71	0.70	0.71
3 (Dependent)	0.70	0.67	0.68	0.67	0.72	0.69	0.70	0.69
6B (Sadistic)	0.69	0.68	0.68	0.68	0.68	0.65	0.66	0.65
I (Schizoid)	0.61	0.59	0.60	0.59	0.59	0.57	0.58	0.57
**Factor 2: Externalizing psychopathology**								
6A (Antisocial)	0.84	0.87	0.88	0.87	0.76	−0.82	−0.82	−0.82
T (Drug dependence)	0.82	0.80	0.78	0.80	0.78	−0.77	−0.74	−0.77
B (Alcohol dependence)	0.77	0.75	0.76	0.75	0.69	−0.68	−0.68	−0.68
7 (Compulsive)	−0.75	−0.60	−0.61	−0.60	−0.81	0.65	0.65	0.65
**Factor 3: General state of psychological disturbances**								
5 (Narcissistic)	0.80	0.60	0.60	0.60	0.81	0.61	0.61	0.61
4 (Histrionic)	0.75	0.61	0.63	0.61	0.73	0.59	0.61	0.59

*PCA, principal component analysis; PAF, principal axis factoring; AF, alpha factoring; ULS, unweighted least squares*.

The second factor—externalizing psychopathology (eigenvalue = 1.899)—modeled for 7.9% of the variance and had four scales with excellent loading, exceeding.55 (Table 5). This factor includes antisocial (6A) loadings ≥-0.82, drug dependence (T) loadings ≥-0.74, alcohol dependence (B) loadings ≥-0.68, and compulsive (7) scales loadings ≥-0.60. The compulsive scale (7) was loaded in the opposite direction across the orthogonal and oblique rotation methods of four extraction methods with the other three scales in the same dimension.

The final factor—general state of psychological disturbances (eigenvalue = 1.7)—accounted for 7.1% of the variance and had two factors with loading exceeding.55 (Table 5). This factor consisted of histrionic (4) loadings ≥0.59, and narcissistic scales (5) loadings ≥0.60 as confirmed by orthogonal and oblique rotation procedures across four extraction methods.

### Construct Equivalence

#### Tucker's Congruence Coefficients for MCMI-III

Appendices 1, 2 present the results of principal component analysis (PCA) with Varimax rotation for the current study. This extraction method is presented and selected in consistent with previous American studies (Craig and Bivens, 1998; Haddy et al., 2005).

#### American Study (1): Haddy et al. (2005)

Tucker's congruence coefficients for MCMI-III were calculated by multiplying each loading in the current study by the corresponding loading in Haddy et al. (2005). Next, the sum of these products was divided by the square root of (the sum of squared loadings for the current study times the sum of squared loading for the other study) [see Appendix 1 for more details of computation, results, and scores of congruence coefficients (Φ)]. Table 6 shows the congruence coefficients (Φ) for our sample factors and those factors in the American Study (Haddy et al., 2005), using the principal component analysis (PCA).

**Table 6:** Coefficients of congruence (Φ) between factors of our sample and American study (Haddy et al., 2005).

**Our sample**	**American study (Haddy et al.**, **2005****)**		
	**1**	**2**	**3**
1	**0.86**	0.66	**0.90**
2	**0.80**	**0.97**	0.57
3	−0.64	−0.07	0.53

*Bold value indicates ≥0.80 refers to constructs similarity of two studies based on TCC*.

The coefficient of congruence (Φ) between both studies varied between good (0.80) and excellent rates (>0.90) for congruent factors. Our first factor [F1, internalizing psychopathology (IN)] is congruent with first and third factors obtained by Haddy et al. (2005). Our second factor [F2, externalizing psychopathology (EX)] is congruent with first and second factors obtained by the same study. Our third factor [F3, psychological disturbances (PD)] did not reach significant congruency with any factors obtained by Haddy et al. (2005).

#### American Study (2): Craig and Bivens (1998)

The calculation of the equivalent results for congruence coefficients among three factors of two studies showed that Factor 1 (F1, internalizing psychopathology) is similar with its general maladjustment (GM) factor extracted by Craig and Bivens (1998). It is related also with the second factor labeled as “paranoid behavior” (PB). The externalizing psychopathology (EX) (F2) in the current study is identical with the antisocial acting out (AA) factor extracted by Craig and Bivens (1998). The third factor of the current study, the psychological disturbances (PD), is negatively related with the first factor for the GM sample.

Tucker's congruence coefficients for MCMI-III is calculated by multiplying each loading in the current study by the corresponding loading in the other study (Craig and Bivens, 1998). Next, the sum of these products was divided by the square root of (the sum of squared loadings for the current study times the sum of squared loading for the other study).

Similarly, Table 7 displays the congruence coefficients (Φ) for our sample factors and those factors in the American Study (Craig and Bivens, 1998), using the principal component analysis (PCA). Appendix 2 presents computation, results, and scores of congruence coefficients (Φ).

**Table 7:** Coefficients of congruence (Φ) between factors of our sample and American study (Craig and Bivens, 1998).

**Our sample**	**American study (Craig and Bivens**, **1998****)**		
	**1**	**2**	**3**
1	**0.82**	**0.93**	0.69
2	0.59	0.68	**0.96**
3	−0.67	−0.02	−0.05

*Bold value indicates ≥0.80 refers to constructs similarity of two studies based on TCC*.

The coefficient of congruence (Φ) between both studies varied between good (0.82) and excellent rate (>0.90) for factor 1: [F1, internalizing psychopathology (IN)] and Factor 2: [F2, externalizing psychopathology (EX)]. Our third factor: psychological disturbances (PD) did not reach significant congruency with any factors obtained by Craig and Bivens (1998).

#### Arabic and Italian Versions of MCM-III

The results of principal component analysis (PCA) with direct oblimin are presented in Appendices 3, 4 are in line with the Italian version study of the MCM-III (Pignolo et al., 2017) that obtained the four-factor model for both linear independent and dependent types of scales of MCM-III. Independent scales of MCMI-III directly reflect DSM-IV diagnostic criteria of disorders and given a weight of two points, whereas dependent scales of MCMI-III refer to items that are more peripheral to the construct, which receives a weight of one point (Craig, 2005).

Table 8 displays the congruence coefficients (Φ) for our sample factors and the factors in the Italian study (Pignolo et al., 2017), using the dependent scales. Appendices 3, 4 present details of results and scores of congruence coefficients (Φ).

**Table 8:** Coefficients of congruence (Φ) between factors of our sample and factors based on linearly dependent scales of Italian study.

**Our sample**	**Italian study (Pignolo et al.**, **2017****)**			
	**1**	**2**	**3**	**4**
1	**0.88**	0.34	0.48	−0.27
2	0.00	**0.86**	0.20	−0.13
3	−0.33	0.07	0.27	0.77

*Bold value indicates ≥0.80 refers to constructs similarity of two studies based on TCC*.

The coefficient of congruence (Φ) between both studies was a good rate (>0.80) for Factor 1: [F1, internalizing psychopathology (IN)] and Factor 2: [F2, externalizing psychopathology (EX)]. Our third factor: psychological disturbances (PD) did not reach significant congruency with any factors established by Pignolo et al. (2017).

Table 9 displays the congruence coefficients (Φ) for our sample factors and factors in the Italian study (Pignolo et al., 2017), using the principal component analysis (PCA) with linearly independent scale. Appendix 4 presented details of results and scores of congruence coefficients (Φ).

**Table 9:** Coefficients of congruence (Φ) between factors of our sample and factors based on linearly independent scales of Italian study.

**Our sample**	**Italian study (Pignolo et al.**, **2017****)**			
	**1**	**2**	**3**	**4**
1	**0.84**	0.63	−0.16	−0.59
2	0.69	0.45	0.30	−0.38
3	−0.30	0.41	0.08	0.59

*Bold value indicates ≥0.80 refers to constructs similarity of two studies based on TCC*.

The coefficient of congruence (Φ) between both studies was a good rate (>0.80) for Factor 1: [F1, internalizing psychopathology (IN)] and Factor 1 extracted by Pignolo et al. (2017). Our second and third factors: [F2, externalizing psychopathology (EX)] and psychological disturbance (PD) did not reach significant congruency with any factors established by Pignolo et al. (2017).

#### Arabic and Dutch Versions of MCM-III

Great work and innovative studies on MCMI-III were conducted by Gina Rossi and her team. The results of principal component analysis (PCA) direct oblimin with a structure matrix presented in Appendices 5, 6 are in line with previous study of the Dutch version of MCM-III (Rossi et al., 2007), which obtained the four-factor model for linear independent and dependent types of scales of MCM-III, presenting only the first one, using both structure and pattern matrix. Appendices 5, 6 presented details of results and scores of congruence coefficients (Φ).

Table 10 shows the correlations among the factors.

**Table 10:** Factor correlation matrix for the structure factor solution.

**Factors**	**F1**	**F2**	**F3**
F1	1	0.654^**^	−0.793^**^
F2	0.654^**^	1	−0.458^*^
F 3	−0.793^**^	−0.458^*^	1

** *Correlation is significant at the 0.01 level (2-tailed)*.

* *Correlation is significant at the 0.05 level (2-tailed)*.

Table 11 shows the congruence coefficients (Φ) for our sample factors and factors based on the Dutch study (Rossi et al., 2007), using the structure analysis. The coefficient of congruence (Φ) between both studies varied from good (0.80) to an excellent rate (>0.90) for Factor 1: [F1, internalizing psychopathology (IN)] and Factor 2: [F2, externalizing psychopathology (EX)] established by Rossi et al. (2007). Our third factor: psychological disturbance did not reach significant congruency with any factors established by Rossi et al. (2007).

**Table 11:** Coefficients of congruence (Φ) between factors of our sample and the Dutch study (Rossi et al., 2007), using the structure matrix.

**Our SAMPLE**	**Dutch study (Rossi et al.**, **2007****)**			
	**1**	**2**	**3**	**4**
1	**0.95**	**0.80**	**0.94**	−0.72
2	0.71	**0.93**	0.56	−0.52
3	−0.28	0.13	0.07	0.65

*Bold value indicates ≥0.80 refers to constructs similarity of two studies based on TCC*.

Table 12 displays the congruence coefficients (Φ) for our sample factors and factors based on the Dutch study (Rossi et al., 2007), using the principal component analysis (PCA). Appendix 6 presents details of results and scores of congruence coefficients (Φ).

**Table 12:** Coefficients of congruence (Φ) between factors of our sample and Dutch study (Rossi et al., 2007), using the principal component analysis (PCA).

**Our sample**	**Dutch study (Rossi et al.**, **2007****)**			
	**1**	**2**	**3**	**4**
1	**0.89**	0.18	0.61	−0.42
2	0.13	0.74	−0.04	−0.04
3	−0.20	0.23	0.35	0.73

*Bold value indicates ≥0.80 refers to constructs similarity of two studies based on TCC*.

The coefficient of congruence (Φ) between both studies was excellent (0.89) for Factor 1: [F1, internalizing psychopathology (IN)] and Factor 1 established by Rossi et al. (2007). Our second and third factors: [F2, externalizing psychopathology (EX)] and psychological disturbance (PD) did not reach significant congruency with any factors established by Rossi et al. (2007).

## Discussion

The first aim of the study is to test the normal distribution of the scales of the MCMI–III. The results of the study demonstrate that the scales of the MCMI–III are normally distributed. Both values of the skewness and kurtosis, and descriptive statistics confirm that 24 scales of MCMI are normally distributed, which is in line with previous study (Millon et al., 2006).

The second aim of the study is to evaluate the internal consistency of MCMI–III. The results of the study indicate each of 24 factors of MCMI–III is reliable cross of the four domains of classification of psychopathology: the 11 clinical personality disorders, the three severe personality disorders, the seven clinical syndromes, and the three severe clinical syndromes. Additionally, the internal consistency of all scales of MCMI–III is ideally rated degree. Values of Cronbach's alpha's reliability in the current study is more satisfactory in comparison with previous studies (Millon et al., 2006). Particularly, Cronbach's alpha for compulsive and narcissistic scales was 0.87 and 0.84, respectively.

Finally, the third aim of the study is to determine the underlying structures of MCMI–III. The results of the study extracted three-factor solutions across eight methods of factor analysis: internalizing psychopathology, externalizing psychopathology, and general state of psychological disturbances.

The first factor—internalizing psychopathology—includes paranoid, anxiety, post-traumatic stress, depressive, thought disorder, schizotypal, dysthymia, avoidant, major depression, borderline, somatoform, delusional disorder, bipolar manic, masochistic, negativistic, dependent, sadistic, and schizoid scales.

All disorders in this factor were centered around self-detachment (Millon et al., 2006), which could be called “internalizing psychopathology”(Krueger et al., 1998, 2001, 2016; Krueger, 1999; Kupfer et al., 2008) “general maladjustment with a psychotic thoughts” factor as discussed in MCMI–III previous studies (Rossi et al., 2007).

The second factor—externalizing psychopathology—includes antisocial, drug dependence, alcohol dependence, and compulsive scales. The compulsive personality disorder was loaded in the opposite direction across the orthogonal and oblique rotation methods of the four extraction methods with the other three disorders in the same factor.

Millon et al. (2006) illustrated that patients with compulsive scale seek to be a fully perfect, ideal man with highly strict values, fearful of social rejection, avoiding behaviors and manners of antisocial personality, and personalities that are addicted to alcohol and drugs. The four scales were centered around detachment and deviation from social values and society. These disorders extracted together in this research as one factor were in line with previous studies on MCMI–III (Rossi et al., 2007), labeling social acting out, social deviance, and, recently, “externalizing psychopathology” (Krueger et al., 1998, 2001, 2016; Krueger, 1999; Kupfer et al., 2008).

The final factor—general state of psychological disturbances—consisted of histrionic personality and narcissistic personality scales. The term of the factor was theoretically supported from the manual of MCMI–III (Millon et al., 2006). Empirically, this term, including histrionic and narcissistic scales, was closely reported in the previous literature on the MCMI–III, using different labels as emotional instability/detachment (Rossi et al., 2007), hostile dominance (Haddy et al., 2005), and classical introversion-extraversion dimension by Sloore et al. (Rossi et al., 2010).

Like original correlation matrix of MCMI–III, the 21 variables were positively correlated in this study, while three variables: histrionic personality, narcissistic personality, and compulsive personality scales were negatively correlated. Correlation matrix in this study is similar to its pair in the MCMI–III manual in terms of direction correlation.

### Construct Equivalence

The relevance of studying construct equivalence for MCMI-III factor structure has been acknowledged by many researchers (Rossi et al., 2007; Pignolo et al., 2017).

The authors compared the results of MCMI-III with the results reported in four previous studies of MCMI-III from the western culture (Craig and Bivens, 1998; Haddy et al., 2005; Rossi et al., 2007; Pignolo et al., 2017) as these studies are characterized by adequate sample size, excellent presentation of the results, thus allowing further *post-hoc* comparison based on Tucker's congruence coefficients (TCC), and have enriching information on MCMI-III in the context of Western culture.

It is remarkable that TCC between the current study and two studies provided by American States in three-factor solutions were higher than 0.80, varying from 0.82 to 0.97. Values of congruence were >0.80 for factors should be evaluated as congruent, suggesting a fair and excellent similarity between the factor structures. Clearly, internalizing and externalizing psychopathology solutions extracted by our data are congruent with extracted solutions obtained by American cultures: high vs. low psychopathology and low vs. high emotional constraint (Haddy et al., 2005), and general maladjustment and antisocial acting out (Craig and Bivens, 1998).

Psychological disturbances in this study were found dissimilar to solutions obtained by American cultures: high vs. low psychopathology (Haddy et al., 2005), and general maladjustment and antisocial acting out (Craig and Bivens, 1998).

Regarding the results of congruence coefficient (Φ) between the three-factors solution and four-factor solution extracted by the Italian and Dutch versions of the MCMI-III, the first two factors (internalizing and externalizing psychopathology solution) are congruent by values above 0.80, which reflect similarities of factors solution between the data of the current study and European (Rossi et al., 2007; Pignolo et al., 2017) versions of MCMI-III. Psychological disturbances did not also reach for an adequately congruent level with European studies (Rossi et al., 2007; Pignolo et al., 2017).

Moreover, psychological disturbances loaded on narcissistic and histrionic scales. Studies on correlations matrix of scales of MCMI-III found the negative relationship between those two disorders and the rest of the scales. This negative direction was rooted up in the theory of Millon of personality that was expressed by MCMI. In addition, a high interrelationship between three-factor solution and four-factor solution extracted by Italian and Dutch versions of MCMI-III was found, reflecting similarities among factor solutions between the data of the current study and European versions of MCMI-III.

Most significantly, the factor structure obtained in this study confirms that the MCMI–III is a multidimensional scale, tapping into the specific dimensions of psychopathology.

The four methods of factor analysis (e.g., PCA, PAF, AF, and ULS with varimax and direct oblimin rotations) confirmed similar results. It was noted that using more than one method of factor analysis can lead to similar results when the theoretical model based on data is well-structured (Goldberg and Digman, 1994; Rossi et al., 2007; Tabachnick et al., 2007). In this study, this statistical idea was found to be true for the factor structure of the MCMI–III, and using several methods in exploratory research is one of the contributions in terms of the methodology. Moreover, Tucker's congruence coefficient was more exhaustive and transparent for detecting the similarity of compared factors across cultures than verbally expressed approach.

It is clear that the current study importantly extends the existing disorders of internalizing psychopathology structure by including a variety of Axes I and II psychopathology. Theoretically, this expanded the conceptualization of internalizing psychopathology, which is consistent with the ideas of Krueger and his team (Krueger et al., 2016) that assume the probability of testing the internalizing model with many mental disorders.

MCMI-III is an advanced step in clinical assessment, capturing several forms of psychopathology or mental disorders detailed in DSM. The researchers, clinicians, and clinical psychologists can use MCMI-III to explore and decide the diagnostic process for internalizing and externalizing constructs of psychopathology in clinical settings. It also can get benefits from the cognitive-behavioral programs and clinical interventions to reduce psychopathological symptoms. In other words, professionals in psychology and public health can use psychological interventions based on cognitive therapy to reduce psychopathological symptoms in a similar way with the Western culture.

It is strongly recommended that researchers and clinicians expand the use of the MCMI–III for identifying the clinically significant dimensions of psychopathology in non-Western culture. Classification of psychopathology in terms of the internalizing-externalizing model is more reasonable than both personality disorders and clinical syndromes.

This study has limitations that should be noted. Since the participants were outpatients, the generalizability of the findings to other populations is questionable. Similarily, the participants were selected from a country of the Arab world, so the generalizability of the findings to other populations is also questionable. The sample size of this study is optimal according to the guideline given by Stevens (2012) 5–20 participants per item (factors in this study); however, 212 patients are still small.

Although the MCMI–III was evident by excellent psychometric properties, using exploratory factor analysis, replication of this study is needed and more plausible, using different methodologies, such as multi-trait multi-methods (e.g., self-reports, clinical observations, and interviews) and samples (e.g., prisoners, patients, and normal population) with optimal sample size. In conclusion, this study is adequately explored factor structure of MCMI–III, using a clinical sample in a non-Western country, which found MCMI–III to be precise and psychometrically credible for screening of psychopathology/mental disorders.

## Data Availability Statement

The original contributions presented in the study are included in the article/Supplementary Material, further inquiries can be directed to the corresponding author/s.

## Ethics Statement

The studies involving human participants were reviewed and approved by Committee of Department of Educational Psychology and Counseling in International Islamic University Malaysia (IIUM), Malaysia. The participants provided their written informed consent to participate in the study.

## Author Contributions

All authors listed have made a substantial, direct and intellectual contribution to the work, and approved it for publication.

## Conflict of Interest

The authors declare that the research was conducted in the absence of any commercial or financial relationships that could be construed as a potential conflict of interest.

## Publisher's Note

All claims expressed in this article are solely those of the authors and do not necessarily represent those of their affiliated organizations, or those of the publisher, the editors and the reviewers. Any product that may be evaluated in this article, or claim that may be made by its manufacturer, is not guaranteed or endorsed by the publisher.

## References

[B1] APA (1994). Diagnostic and Statistical Manual of Mental Disorders. Washington, DC, American Psychiatric Publishing.

[B2] APA (2000). Diagnostic and Statistical Manual of Mental Disorders. Washington, DC: American Psychiatric Association.

[B3] APA (2013). Diagnostic and statistical manual of mental disorders (DSM-5^®^): American Washington, DC: Psychiatric Pub.

[B4] Boccaccini M. T. Brodsky S. L. (2002). Attorney–client trust among convicted criminal defendants: preliminary examination of the attorney–client trust scale. Behav. Sci. Law 20, 69–87. 10.1002/bsl.469 11979492

[B5] Butcher J. N. Hamilton C. K. Rouse S. V. Cumella E. J. (2006). The deconstruction of the Hy scale of MMPI−2: failure of RC3 in measuring somatic symptom expression. J. Pers. Assess. 87, 186–192. 10.1207/s15327752jpa8702_08 16972822

[B6] Craig R. J. (ed.). (2005). New Directions in Interpreting the Millon Clinical Multiaxial Inventory-III (MCMI-III). John Wiley & Sons.

[B7] Craig R. J. Bivens A. (1998). Factor structure of the MCMI-III. J. Pers. Assess. 70, 190–196. 10.1207/s15327752jpa7001_13 9615431

[B8] Davidov E. Meuleman B. Cieciuch J. Schmidt P. Billiet J. (2014). Measurement equivalence in cross-national research. Annu. Rev. Sociol. 40, 55–75. 10.1146/annurev-soc-071913-043137

[B9] Dyce J. A. O'Connor B. P. Parkins S. Y. Janzen H. L. (1997). Correlational structure of the MCMI-III personality disorder scales and comparisons with other data sets. J. Pers. Assess. 69, 568–582. 10.1207/s15327752jpa6903_10 9501485

[B10] Egger J. De Mey H. Derksen J. Van Der Staak C. (2003). MMPI-2 and MCMI-III scores among Dutch inpatient substance abusers: assessing correspondence and cross-cultural equivalence. Curr. Psychol. 22, 117–124. 10.1007/s12144-003-1002-x

[B11] Exner J. (1993). The Rorschach: A Comprehensive System, Vol. 1. 3rd Edn. New York, NY: Wiley.

[B12] Field A. (2013). Discovering Statistics Using IBM SPSS Statistics. London: sage.

[B13] Goldberg L. R. Digman J. M. (1994). Revealing structure in the data: Principles of exploratory factor analysis, in Differentiating Normal and Abnormal Personality, eds Strack S. Lorr M. (Springer Publishing Company), 216–242.

[B14] Gravetter F. J. Wallnau L. B. Forzano L.-A. B. (2016). Essentials of Statistics for the Behavioral Sciences.

[B15] Grossman S. D. (2004). Facets of Personality: A Proposal for the Development of MCMI-III Content Scales. Carlos Albizu University.

[B16] Haddy C. Strack S. Choca J. P. (2005). Linking personality disorders and clinical syndromes on the MCMI-III. J. Pers. Assess. 84, 193–204. 10.1207/s15327752jpa8402_09 15799894

[B17] Hair J. Black W. Babin B. Anderson R. (2009). Exploratory Factor Analysis Multivariate Data Analysis. Hoboken, NJ: Prentice Hall.

[B18] Hsu L. (2002). Diagnostic validity statistics and the MCMI-III. Psychol. Assess. 14:410. 10.1037/1040-3590.14.4.410 12501567

[B19] Hsu L. (2005). Using critiques of the MCMI to improve MCMI research and interpretations, in New Directions in Interpreting the Millon™ Clinical Multiaxial Inventory-III (MCMI-III™), ed Craig R. J. (John Wiley & Sons Inc), 290–320.

[B20] Krueger R. F. (1999). The structure of common mental disorders. Arch. Gen. Psychiatry 56, 921–926. 10.1001/archpsyc.56.10.921 10530634

[B21] Krueger R. F. Caspi A. Moffitt T. E. Silva P. A. (1998). The structure and stability of common mental disorders (DSM-III-R): a longitudinal-epidemiological study. J. Abnorm. Psychol. 107, 216–227. 10.1037/0021-843X.107.2.216 9604551

[B22] Krueger R. F. McGue M. Iacono W. G. (2001). The higher-order structure of common DSM mental disorders: internalization, externalization, and their connections to personality. Pers. Individ. Dif. 30, 1245–1259. 10.1016/S0191-8869(00)00106-9

[B23] Krueger R. F. Tackett J. L. MacDonald A. (2016). Toward validation of a structural approach to conceptualizing psychopathology: a special section of the journal of abnormal psychology. J. Abnorm. Psychol. 125, 1023–1026. 10.1037/abn0000223 27819464

[B24] Kupfer D. J. First M. B. Regier D. A. (2008). A Research Agenda for DSM V. Washington, DC: American Psychiatric Pub.

[B25] Leech N. L. Barrett K. C. Morgan G. A. (2015). IBM SPSS for Intermediate Statistics: Use and Interpretation, 5th Edn. Routledge/Taylor & Francis Group.

[B26] Lorenzo-Seva U. Ten Berge J. M. (2006). Tucker's congruence coefficient as a meaningful index of factor similarity. Methodology 2, 57–64. 10.1027/1614-2241.2.2.57

[B27] Millon T. (1981). Disorders of Personality: DSM-III, Axis II. John Wiley & Sons.

[B28] Millon T. (1983). Modern Psychopathology: A Biosocial Approach to Maladaptive Learning and functioning. Waveland PressInc.

[B29] Millon T. (1987). Millon Clinical Multiaxial Inventory II. Ncs interpretive scoring systems.

[B30] Millon T. (1990). Toward a New Personology: An Evolutionary Model. New York, NY: John Wiley & Sons.

[B31] Millon T. (1994). MCMI-III Manual: Millon Clinical Multiaxial Inventory–II. Minneapolis, MN: National Computer Systems.

[B32] Millon T. Davis R. D. Millon C. (1997). MCMI-III Manual. National Computer Systems.

[B33] Millon T. Millon C. Davis R. D. Grossman S. D. (2006). MCMI-III Manual, 3 Edn. Minneapolis, MN: Pearson Education, Inc.

[B34] Millon T. Millon C. M. Meagher S. E. Grossman S. D. Ramnath R. (2004). Personality Disorders in Modern Life. New York, NY: John Wiley & Sons

[B35] Millon T. Millon C. M. Meagher S. E. Grossman S. D. Ramnath R. (2012). Personality Disorders in Modern Life. New York, NY: John Wiley and Sons.

[B36] Millon T. E. (1997). The Millon Inventories: Clinical and Personality Assessment. The Guilford Press.

[B37] Nasiri H. Abedi A. Ebrahimi A. Ameli S. S. Samouei R. (2013). Personality profile of women affected with borderline personality disorder. Mater. Sociomed. 25, 60–63. 10.5455/msm.2013.25.60-63 23687463 PMC3655790

[B38] Nunnally J. C. (1978). Psychometric Theory, 2 Edn. New York, NY: McGraw-Hill.

[B39] Pignolo C. Rosalo R. Andò A. Cristofanelli S. Ferro L. Zennaro A. (2017). The factor structure of the italian version of the MCMI-III compared to the Dutch and American versions. BPA Appl. Psychol. Bull. 65, 36–46.

[B40] Piotrowski C. Lubin B. (1990). Assessment practices of health psychologists: survey of APA division 38 clinicians. Prof. Psychol. Res. Pract. 21, 99–106. 10.1037/0735-7028.21.2.99

[B41] Rossi G. Derksen J. (2015). International adaptations of the millon clinical multiaxial inventory: construct validity and clinical applications. J. Pers. Assess. 97, 572–590. 10.1080/00223891.2015.1079531 26473456

[B42] Rossi G. Elklit A. Simonsen E. (2010). Empirical evidence for a four factor framework of personality disorder organization: multigroup confirmatory factor analysis of the millon clinical multiaxial inventory—III personality disorder scales across belgian and danish data samples. J. Pers. Disord. 24, 128–150. 10.1521/pedi.2010.24.1.128 20205502

[B43] Rossi G. van der Ark L. A. Sloore H. (2007). Factor analysis of the Dutch-language version of the MCMI–III. J. Pers. Assess. 88, 144–157. 10.1080/00223890701267977 17437380

[B44] Segal D. L. Coolidge F. L. Rosowsky E. (2006). Personality Disorders and Older Adults: Diagnosis, Assessment, and Treatment. John Wiley & Sons Inc.

[B45] Stevens J. P. (2012). Applied Multivariate Statistics for the Social Sciences. Routledge. 10.4324/9780203843130

[B46] Strack S. (2002). Essentials of Millon Inventories Assessment, 2nd Edn. New York: John Wiley & Sons. 1–51.

[B47] Strack S. Millon T. (2007). Contributions to the dimensional assessment of personality disorders using Millon's model and the Millon clinical multiaxial inventory (MCMI–III). J. Pers. Assess. 89, 56–69. 10.1080/00223890701357217 17604534

[B48] Tabachnick B. G. Fidell L. S. Ullman J. B. (2007). Using Multivariate Statistics (Vol. 5). Boston, MA: Pearson. 481–498.

[B49] Ten Berge J. M. (1986). Rotation to perfect congruence and the cross validation of component weights across populations. Multivariate Behav. Res. 21, 41–64. 26760919 10.1207/s15327906mbr2101_3

[B50] Thompson B. (2004). Exploratory and Confirmatory Factor Analysis: Understanding Concepts and Applications. Washington, DC: American Psychological Association. 10.1037/10694-000

[B51] Trochim W. (2006). Research Methods Knowledge Base. Web Centre for social research methods. Available online at: http://www.socialresearchmethods.net/kb/ (accessed March 14, 2015).

[B52] Tucker L. R. (1951). A Method for Synthesis of Factor Analysis Studies. Princeton, NJ: Educational testing service.

[B53] Van de Vijver F. Tanzer N. K. (2004). Bias and equivalence in cross-cultural assessment: an overview. Euro. Rev. Appl. Psychol. 54, 119–135. 10.1016/j.erap.2003.12.004

[B54] Ward L. C. (1995). Correspondence of the MMPI-2 and MCMI-II in male substance abusers. J. Pers. Assess. 64, 390–393. 10.1207/s15327752jpa6402_18 7722864

