# Supplementary material for: Psychometric Properties of the Millon Clinical Multiaxial Inventory–III in an Arabic Clinical Sample Compared With American, Italian, and Dutch Cultures
Source: Front Psychol. 2021 Sep 9;12:562619. doi: 10.3389/fpsyg.2021.562619 (PMC8458952; doi:10.3389/fpsyg.2021.562619)
Supplement: Supplementary file 1 [file Table_1.docx]

**Appendix 1: PCA Results of Current Study and American Study**

| **Scales** | **Arabic Study** | | | **American Study** | | | **Tucker’s Congruence Coefficients (TCC)** | | | | | | | | |
| --- | --- | --- | --- | --- | --- | --- | --- | --- | --- | --- | --- | --- | --- | --- | --- |
|  | **Current Study** | | | **Haddy, Strack and**  **Choca (2005)** | | |  |  |  |  |  |  |  |  |  |
|  | F1IN | F2EX | F3PD | F1HLP | F2LHE | F3PT | **F1*1** | **F1*2** | **F1*3** | **F2*1** | **F2*2** | **F2*3** | **F3*1** | **F3*2** | **F3*3** |
| I (Schizoid) | .607 | .436 | -.313 | .64 | .14 | .22 | .39 | .08 | .13 | .28 | .06 | .10 | -.20 | -.04 | -.07 |
| 2A (Avoidant) | .812 | .135 | -.221 | .74 | .14 | .25 | .60 | .11 | .20 | .10 | .02 | .03 | -.16 | -.03 | -.06 |
| 2B (Depressive) | .818 | .251 | -.210 | .74 | .28 | .29 | .61 | .23 | .24 | .19 | .07 | .07 | -.16 | -.06 | -.06 |
| 3 (Dependent) | .701 | .246 | -.052 | .64 | .15 | .24 | .45 | .11 | .17 | .16 | .04 | .06 | -.03 | -.01 | -.01 |
| 4 (Histrionic) | -.274 | -.066 | .749 | -.79 | -.15 | -.03 | .22 | .04 | .01 | .05 | .01 | .00 | -.59 | -.11 | -.02 |
| 5 (Narcissistic) | .066 | .060 | .799 | -.77 | -.01 | .34 | -.05 | .00 | .02 | -.05 | .00 | .02 | -.62 | -.01 | .27 |
| 6A (Antisocial) | .409 | .839 | .135 | .12 | .92 | .17 | .05 | .38 | .07 | .10 | .77 | .14 | .02 | .12 | .02 |
| 6B (Sadistic) | .690 | .453 | .183 | .17 | .64 | .49 | .12 | .44 | .34 | .08 | .29 | .22 | .03 | .12 | .09 |
| 7 (Compulsive) | .015 | -.751 | .305 | -.48 | -.64 | -.03 | -.01 | -.01 | .00 | .36 | .48 | .02 | -.15 | -.20 | -.01 |
| 8A (Negativistic) | .715 | .297 | .011 | .51 | .37 | .56 | .36 | .26 | .40 | .15 | .11 | .17 | .01 | .00 | .01 |
| 8B (Masochistic) | .722 | .475 | -.093 | .62 | .36 | .29 | .45 | .26 | .21 | .29 | .17 | .14 | -.06 | -.03 | -.03 |
| S (Schizotypal) | .815 | .258 | -.076 | .60 | .21 | .54 | .49 | .17 | .44 | .15 | .05 | .14 | -.05 | -.02 | -.04 |
| C (Borderline) | .765 | .478 | -.024 | .58 | .56 | .36 | .44 | .43 | .28 | .28 | .27 | .17 | -.01 | -.01 | -.01 |
| P (Paranoid) | .840 | -.068 | .189 | .34 | .15 | .76 | .29 | .13 | .64 | -.02 | -.01 | -.05 | .06 | .03 | .14 |
| A (Anxiety) | .840 | .202 | -.015 | .60 | .23 | .48 | .50 | .19 | .40 | .12 | .05 | .10 | -.01 | .00 | -.01 |
| H (Somatoform) | .759 | .219 | -.176 | .64 | .03 | .24 | .49 | .02 | .18 | .14 | .01 | .05 | -.11 | -.01 | -.04 |
| N (Bipolar ):Manic | .730 | .374 | .049 | .18 | .44 | .58 | .13 | .32 | .42 | .07 | .16 | .22 | .01 | .02 | .03 |
| D (Dysthymia) | .812 | .291 | -.190 | .79 | .24 | .26 | .64 | .19 | .21 | .23 | .07 | .08 | -.15 | -.05 | -.05 |
| B (Alcohol Dependence) | .439 | .773 | .034 | .18 | .81 | .11 | .08 | .36 | .05 | .14 | .63 | .09 | .01 | .03 | .00 |
| T (Drug Dependence) | .303 | .824 | .112 | .06 | .87 | .13 | .02 | .26 | .04 | .05 | .72 | .11 | .01 | .10 | .01 |
| R (Posttraumatic Stress) | .823 | .210 | -.085 | .66 | .27 | .42 | .54 | .22 | .35 | .14 | .06 | .09 | -.06 | -.02 | -.04 |
| SS (Thought Disorder) | .818 | .351 | -.125 | .65 | .31 | .46 | .53 | .25 | .38 | .23 | .11 | .16 | -.08 | -.04 | -.06 |
| CC (Major Depression) | .805 | .273 | -.175 | .75 | .13 | .22 | .60 | .10 | .18 | .20 | .04 | .06 | -.13 | -.02 | -.04 |
| PP (Delusional Disorder) | .754 | .174 | .261 | .13 | .11 | .80 | .10 | .08 | .60 | .02 | .02 | .14 | .03 | .03 | .21 |
| Sum |  |  |  |  |  |  | **8.04** | **4.65** | **5.95** | **3.46** | **4.18** | **2.32** | **-2.39** | **-.21** | **.25** |

∑X^2^/ Sum of the squared loadings for our study: F1=11.22, F2 = 4.29, and F3 =1.79

∑Y^2^/ Sum of the squared loadings for Haddy, Strack and Choca’ s study (2007): F1=7.76, F2 = 4.39, and F3 = 3.83

We applied congruence coefficient (Φ) formula (p.11), obtaining a series of outcome inserted in Table 6.

$f1f1=\frac{8.04}{\sqrt{11.22(7.76)}}=0.86$ $f1f2=\frac{4.65}{\sqrt{11.22(4.39)}}=0.66$

$$f1f3=\frac{5.95}{\sqrt{11.22(3.83)}}=0.90$$

$$f2f1=\frac{3.46}{\sqrt{4.29(4.39)}}=0.80$$

$$f2f2=\frac{4.18}{\sqrt{4.29(4.39)}}=0.97$$

$$f2f3=\frac{2.32}{\sqrt{4.29(3.83)}}=0.57$$

$$f3f1=\frac{-2.39}{\sqrt{1.79(7.76)}}=-0.64$$

$$f3f2=\frac{-.21}{\sqrt{1.79(4.39)}}=-0.07$$

$$f3f3=\frac{.25}{\sqrt{1.79(3.83)}}=0.53$$

**Appendix 2. PCA Results of Current Study and American Study**

| **Scales** | **Arabic Study** | | | **American Study** | | | **Tucker’s Congruence Coefficients (TCC)** | | | | | | | | |
| --- | --- | --- | --- | --- | --- | --- | --- | --- | --- | --- | --- | --- | --- | --- | --- |
|  | **Current Study** | | | **Craig and Bivens**  **(1998)** | | |  |  |  |  |  |  |  |  |  |
|  | F1IN | F2EX | F3PD | F1 GM | F2 PB | F3 AA | **F1*1** | **F1*2** | **F1*3** | **F2*1** | **F2*2** | **F2*3** | **F3*1** | **F3*2** | **F3*3** |
| I (Schizoid) | .607 | .436 | -.313 | 0.61 | 0.29 | 0.07 | **.37** | **.18** | **.04** | **.27** | **.13** | **.03** | **-.19** | **-.09** | **-.02** |
| 2A (Avoidant) | .812 | .135 | -.221 | 0.67 | 0.42 | 0.12 | **.54** | **.34** | **.10** | **.09** | **.06** | **.02** | **-.15** | **-.09** | **-.03** |
| 2B (Depressive) | .818 | .251 | -.210 | 0.72 | 0.34 | 0.26 | **.59** | **.28** | **.21** | **.18** | **.09** | **.07** | **-.15** | **-.07** | **-.05** |
| 3 (Dependent) | .701 | .246 | -.052 | 0.55 | 0.41 | 0.20 | **.39** | **.29** | **.14** | **.14** | **.10** | **.05** | **-.03** | **-.02** | **-.01** |
| 4 (Histrionic) | -.274 | -.066 | .749 | -0.74 | -0.15 | -0.07 | **.20** | **.04** | **.02** | **.05** | **.01** | **.00** | **-.55** | **-.11** | **-.05** |
| 5 (Narcissistic) | .066 | .060 | .799 | -0 .77 | 0.32 | 0.03 | **-.05** | **.02** | **.00** | **-.05** | **.02** | **.00** | **-.62** | **.26** | **.02** |
| 6A (Antisocial) | .409 | .839 | .135 | 0.05 | 0.09 | 0.88 | **.02** | **.04** | **.36** | **.04** | **.08** | **.74** | **.01** | **.01** | **.12** |
| 6B (Sadistic) | .690 | .453 | .183 | 0.1 | 0.39 | 0.68 | **.07** | **.27** | **.47** | **.05** | **.18** | **.31** | **.02** | **.07** | **.12** |
| 7 (Compulsive) | .015 | -.751 | .305 | -0.5 | -0_,_17 | -0.53 | **-.01** | **.00** | **-.01** | **.38** | **.13** | **.40** | **-.15** | **-.05** | **-.16** |
| 8A (Negativistic) | .715 | .297 | .011 | 0.45 | 0.56 | 0.39 | **.32** | **.40** | **.28** | **.13** | **.17** | **.12** | **.00** | **.01** | **.00** |
| 8B (Masochistic) | .722 | .475 | -.093 | 0.55 | 0.31 | 0.37 | **.40** | **.22** | **.27** | **.26** | **.15** | **.18** | **-.05** | **-.03** | **-.03** |
| S (Schizotypal) | .815 | .258 | -.076 | 0. 51 | 0.65 | 0.17 | **.10** | **.53** | **.14** | **.03** | **.17** | **.04** | **-.01** | **-.05** | **-.01** |
| C (Borderline) | .765 | .478 | -.024 | 0.53 | 0.43 | 0.56 | **.41** | **.33** | **.43** | **.25** | **.21** | **.27** | **-.01** | **-.01** | **-.01** |
| P (Paranoid) | .840 | -.068 | .189 | 0.30 | 0.79 | 0.04 | **.25** | **.66** | **.03** | **-.02** | **-.05** | **.00** | **.06** | **.15** | **.01** |
| A (Anxiety) | .840 | .202 | -.015 | 0.45 | 0.55 | 0.32 | **.38** | **.46** | **.27** | **.09** | **.11** | **.06** | **-.01** | **-.01** | **.00** |
| H (Somatoform) | .759 | .219 | -.176 | 0.63 | 0.31 | 0.12 | **.48** | **.24** | **.09** | **.14** | **.07** | **.03** | **-.11** | **-.05** | **-.02** |
| N (Bipolar ):Manic | .730 | .374 | .049 | 0.11 | 0.61 | 0.43 | **.08** | **.45** | **.31** | **.04** | **.23** | **.16** | **.01** | **.03** | **.02** |
| D (Dysthymia) | .812 | .291 | -.190 | 0.73 | 0.29 | 0.33 | **.59** | **.24** | **.27** | **.21** | **.08** | **.10** | **-.14** | **-.06** | **-.06** |
| B (Alcohol Dependence) | .439 | .773 | .034 | 0.19 | 0.16 | 0.77 | **.08** | **.07** | **.34** | **.15** | **.12** | **.60** | **.01** | **.01** | **.03** |
| T (Drug Dependence) | .303 | .824 | .112 | 0.12 | -0.02 | 0.77 | **.04** | **-.01** | **.23** | **.10** | **-.02** | **.63** | **.01** | **.00** | **.09** |
| R (Posttraumatic Stress) | .823 | .210 | -.085 | 0.57 | 0.51 | 0.3 | **.47** | **.42** | **.25** | **.12** | **.11** | **.06** | **-.05** | **-.04** | **-.03** |
| SS (Thought Disorder) | .818 | .351 | -.125 | 0.57 | 0.56 | 0.28 | **.47** | **.46** | **.23** | **.20** | **.20** | **.10** | **-.07** | **-.07** | **-.04** |
| CC (Major Depression) | .805 | .273 | -.175 | 0.71 | 0.33 | 0.19 | **.57** | **.27** | **.15** | **.19** | **.09** | **.05** | **-.12** | **-.06** | **-.03** |
| PP (Delusional Disorder) | .754 | .174 | .261 | 0.14 | 0.83 | 0.07 | **.11** | **.63** | **.05** | **.02** | **.14** | **.01** | **.04** | **.22** | **.02** |
| Sum |  |  |  |  |  |  | **6.86** | **6.81** | **4.68** | **3.06** | **2.55** | **4.02** | **-2.27** | **-.07** | **-.14** |

∑X^2^/ Sum of the squared loadings for our study: F1=11.22, F2 = 4.29, and F3 =1.79

∑Y^2^/ Sum of the squared loadings for Craig and Bivens’s study (1998): F1 = 6.30, F2 = 4.75, and F3= 4.09

We applied congruence coefficient (Φ) formula, obtaining a series of outcome inserted in Table 7.

**Appendix 3. PCA Results of Current Study and Italian Study with Linearly Dependent Scales**

| **Scales** | **Arabic Study** | | | **Italian Study** | | | | **Tucker’s Congruence Coefficients (TCC)** | | | | | | | | | | | |
| --- | --- | --- | --- | --- | --- | --- | --- | --- | --- | --- | --- | --- | --- | --- | --- | --- | --- | --- | --- |
|  | **Current Study** | | | (Pignolo et al., 2017) | | | |  |  |  |  |  |  |  |  |  |  |  |  |
|  | F1IN | F2EX | F3PD | **F1** | **F2** | **F3** | **F4** | **F1F1** | **F1F2** | **F1F3** | **F1F4** | **F2F1** | **F2F2** | **F2F3** | **F2F4** | **F3F1** | **F3F2** | **F3F3** | **F3F4** |
| I (Schizoid) | .607 | .436 | -.313 | .31 | .04 | .30 | -.49 | .19 | .02 | .18 | -.30 | .13 | .02 | .13 | -.21 | -.10 | -.01 | -.09 | .15 |
| 2A (Avoidant) | .812 | .135 | -.221 | .29 | .08 | .30 | -.83 | .24 | .07 | .24 | -.67 | .04 | .01 | .04 | -.11 | -.06 | -.02 | -.07 | .18 |
| 2B (Depressive) | .818 | .251 | -.210 | .84 | .02 | -.01 | -.12 | .69 | .01 | -.01 | -.10 | .21 | .00 | .00 | -.03 | -.18 | .00 | .00 | .03 |
| 3 (Dependent) | .701 | .246 | -.052 | .67 | .03 | .03 | -.19 | .47 | .02 | .02 | -.13 | .16 | .01 | .01 | -.05 | -.03 | .00 | .00 | .01 |
| 4 (Histrionic) | -.274 | -.066 | .749 | .01 | -.09 | -.11 | .89 | .00 | .02 | .03 | -.24 | .00 | .01 | .01 | -.06 | .01 | -.07 | -.08 | .67 |
| 5 (Narcissistic) | .066 | .060 | .799 | -.21 | .13 | .39 | .61 | -.01 | .01 | .03 | .04 | -.01 | .01 | .02 | .04 | -.17 | .11 | .31 | .49 |
| 6A (Antisocial) | .409 | .839 | .135 | .00 | .93 | .12 | .00 | .00 | .38 | .05 | .00 | .00 | .78 | .10 | .00 | .00 | .13 | .02 | .00 |
| 6B (Sadistic) | .690 | .453 | .183 | .22 | .47 | .31 | .11 | .15 | .32 | .21 | .08 | .10 | .21 | .14 | .05 | .04 | .09 | .06 | .02 |
| 7 (Compulsive) | .015 | -.751 | .305 | -.01 | -.63 | .20 | .02 | .00 | -.01 | .00 | .00 | .01 | .47 | -.15 | -.02 | .00 | -.19 | .06 | .01 |
| 8A (Negativistic) | .715 | .297 | .011 | .63 | .16 | .14 | .01 | .45 | .12 | .10 | .01 | .19 | .05 | .04 | .00 | .01 | .00 | .00 | .00 |
| 8B (Masochistic) | .722 | .475 | -.093 | .60 | .12 | .14 | -.25 | .43 | .09 | .10 | -.18 | .28 | .06 | .06 | -.12 | -.06 | -.01 | -.01 | .02 |
| S (Schizotypal) | .815 | .258 | -.076 | .48 | .04 | .47 | -.18 | .39 | .03 | .39 | -.15 | .12 | .01 | .12 | -.05 | -.04 | .00 | -.04 | .01 |
| C (Borderline) | .765 | .478 | -.024 | .80 | .28 | -.51 | .02 | .61 | .21 | -.39 | .02 | .38 | .13 | -.24 | .01 | -.02 | -.01 | .01 | .00 |
| P (Paranoid) | .840 | -.068 | .189 | .10 | -.01 | .86 | -.14 | .08 | -.01 | .72 | -.12 | -.01 | .00 | -.06 | .01 | .02 | .00 | .16 | -.03 |
| A (Anxiety) | .840 | .202 | -.015 | .83 | -.04 | .12 | .05 | .70 | -.03 | .10 | .04 | .17 | -.01 | .02 | .01 | -.01 | .00 | .00 | .00 |
| H (Somatoform) | .759 | .219 | -.176 | .86 | -.13 | .00 | .02 | .66 | -.10 | .00 | .02 | .19 | -.03 | .00 | .01 | -.15 | .02 | .00 | .00 |
| N (Bipolar ):Manic | .730 | .374 | .049 | .44 | .22 | .30 | .39 | .32 | .16 | .22 | .28 | .16 | .08 | .11 | .15 | .02 | .01 | .01 | .02 |
| D (Dysthymia) | .812 | .291 | -.190 | .95 | -.02 | -.14 | -.02 | .77 | -.02 | -.11 | -.02 | .28 | -.01 | -.04 | -.01 | -.18 | .00 | .03 | .00 |
| B (Alcohol Dependence) | .439 | .773 | .034 | .12 | .65 | .10 | -.06 | .05 | .28 | .05 | -.03 | .09 | .50 | .08 | -.05 | .00 | .02 | .00 | .00 |
| T (Drug Dependence) | .303 | .824 | .112 | -.12 | .74 | .08 | .01 | -.04 | .22 | .03 | .00 | -.10 | .61 | .07 | .00 | -.01 | .08 | .01 | .00 |
| R (Posttraumatic Stress) | .823 | .210 | -.085 | .80 | -.02 | .13 | .04 | .66 | -.02 | .10 | .03 | .17 | .00 | .03 | .01 | -.07 | .00 | -.01 | .00 |
| SS (Thought Disorder) | .818 | .351 | -.125 | .91 | .05 | -.01 | .02 | .75 | .04 | -.01 | .02 | .32 | .02 | .00 | .01 | -.11 | -.01 | .00 | .00 |
| CC (Major Depression) | .805 | .273 | -.175 | .91 | -.02 | -.07 | -.08 | .73 | -.02 | -.06 | -.06 | .25 | -.01 | -.02 | -.02 | -.16 | .00 | .01 | .01 |
| PP (Delusional Disorder) | .754 | .174 | .261 | .03 | .05 | .79 | .02 | .02 | .04 | .60 | .02 | .00 | .01 | .14 | .00 | .01 | .01 | .21 | .01 |
| Sum |  |  |  |  |  |  | | **8.29** | **1.86** | **2.59** | **-1.45** | **3.14** | **2.94** | **.61** | **-.42** | **-1.25** | **.16** | **.59** | **1.60** |

∑X^2^/ Sum of the squared loadings for our study: F1=11.22, F2 = 4.29, and F3 =1.79

∑Y^2^/ Sum of the squared loadings for Pignolo et al. (2017): F1 = 7.90, F2 = 2.68, F3= 2.55, and F4=2.44

We applied congruence coefficient (Φ) formula, obtaining a series of outcome inserted in Table 8.

**Appendix 4. PCA Results of Current Study and Italian Study on Linearly Independent Scales**

| **Scales** | **Arabic Study** | | | **Italian Study** | | | | **Tucker’s Congruence Coefficients (TCC)** | | | | | | | | | | | |
| --- | --- | --- | --- | --- | --- | --- | --- | --- | --- | --- | --- | --- | --- | --- | --- | --- | --- | --- | --- |
|  | **Current Study** | | | (Pignolo et al., 2017) | | | |  |  |  |  |  |  |  |  |  |  |  |  |
|  | F1IN | F2EX | F3PD | **F1** | **F2** | **F3** | **F4** | **F1F1** | **F1F2** | **F1F3** | **F1F4** | **F2F1** | **F2F2** | **F2F3** | **F2F4** | **F3F1** | **F3F2** | **F3F3** | **F3F4** |
| I (Schizoid) | .607 | .436 | -.313 | .30 | .22 | -.14 | -.370 | .18 | .13 | -.08 | -.22 | .13 | .09 | -.06 | -.16 | -.09 | -.07 | .04 | .12 |
| 2A (Avoidant) | .812 | .135 | -.221 | .26 | .35 | -.07 | -.500 | .21 | .28 | -.06 | -.41 | .03 | .05 | -.01 | -.07 | -.06 | -.08 | .02 | .11 |
| 2B (Depressive) | .818 | .251 | -.210 | .62 | .15 | -.12 | -.200 | .51 | .12 | -.10 | -.16 | .16 | .04 | -.03 | -.05 | -.13 | -.03 | .03 | .04 |
| 3 (Dependent) | .701 | .246 | -.052 | .54 | .11 | -.80 | -.150 | .38 | .08 | -.56 | -.11 | .13 | .03 | -.20 | -.04 | -.03 | -.01 | .04 | .01 |
| 4 (Histrionic) | -.274 | -.066 | .749 | -.01 | .19 | -.10 | .722 | .00 | -.05 | .03 | -.20 | .00 | -.01 | .01 | -.05 | -.01 | .14 | -.07 | .54 |
| 5 (Narcissistic) | .066 | .060 | .799 | .01 | .62 | .07 | .166 | .00 | .04 | .00 | .01 | .00 | .04 | .00 | .01 | .01 | .49 | .05 | .13 |
| 6A (Antisocial) | .409 | .839 | .135 | .07 | .46 | .32 | .044 | .03 | .19 | .13 | .02 | .06 | .39 | .27 | .04 | .01 | .06 | .04 | .01 |
| 6B (Sadistic) | .690 | .453 | .183 | .01 | .54 | -.02 | .034 | .01 | .38 | -.01 | .02 | .00 | .25 | -.01 | .02 | .00 | .10 | .00 | .01 |
| 7 (Compulsive) | .015 | -.751 | .305 | .07 | .15 | -.44 | .023 | .00 | .00 | -.01 | .00 | -.05 | -.11 | .33 | -.02 | .02 | .05 | -.13 | .01 |
| 8A (Negativistic) | .715 | .297 | .011 | .44 | .35 | -.19 | -.060 | .32 | .25 | -.14 | -.04 | .13 | .10 | -.06 | -.02 | .00 | .00 | .00 | .00 |
| 8B (Masochistic) | .722 | .475 | -.093 | .38 | .34 | .02 | -.260 | .27 | .25 | .01 | -.19 | .18 | .16 | .01 | -.12 | -.04 | -.03 | .00 | .02 |
| S (Schizotypal) | .815 | .258 | -.076 | .31 | .58 | .00 | -.140 | .25 | .47 | .00 | -.11 | .08 | .15 | .00 | -.04 | -.02 | -.04 | .00 | .01 |
| C (Borderline) | .765 | .478 | -.024 | .68 | .15 | .08 | -.500 | .52 | .11 | .06 | -.38 | .33 | .07 | .04 | -.24 | -.02 | .00 | .00 | .01 |
| P (Paranoid) | .840 | -.068 | .189 | -.01 | .78 | -.09 | -.170 | -.01 | .65 | -.08 | -.14 | .00 | -.05 | .01 | .01 | .00 | .15 | -.02 | -.03 |
| A (Anxiety) | .840 | .202 | -.015 | .72 | .16 | .03 | -.060 | .60 | .13 | .03 | -.05 | .14 | .03 | .01 | -.01 | -.01 | .00 | .00 | .00 |
| H (Somatoform) | .759 | .219 | -.176 | .80 | -.08 | .02 | -.040 | .60 | -.06 | .02 | -.03 | .17 | -.02 | .00 | -.01 | -.14 | .01 | .00 | .01 |
| N (Bipolar ):Manic | .730 | .374 | .049 | .07 | .54 | -.24 | -.380 | .05 | .40 | -.18 | -.28 | .03 | .20 | -.09 | -.14 | .00 | .03 | -.01 | -.02 |
| D (Dysthymia) | .812 | .291 | -.190 | .89 | -.17 | -.06 | -.020 | .72 | -.14 | -.05 | -.02 | .26 | -.05 | -.02 | -.01 | -.17 | .03 | .01 | .00 |
| B (Alcohol Dependence) | .439 | .773 | .034 | .12 | .10 | .37 | -.020 | .05 | .04 | .16 | -.01 | .09 | .08 | .29 | -.02 | .00 | .00 | .01 | .00 |
| T (Drug Dependence) | .303 | .824 | .112 | -.01 | .12 | .57 | -.020 | .00 | .03 | .17 | -.01 | -.01 | .09 | .47 | -.02 | .00 | .01 | .06 | .00 |
| R (Posttraumatic Stress) | .823 | .210 | -.085 | .50 | .21 | .10 | -.060 | .41 | .17 | .08 | -.05 | .11 | .04 | .02 | -.01 | -.04 | -.02 | -.01 | .01 |
| SS (Thought Disorder) | .818 | .351 | -.125 | .73 | .10 | -.50 | -.030 | .60 | .08 | -.41 | -.02 | .26 | .04 | -.18 | -.01 | -.09 | -.01 | .06 | .00 |
| CC (Major Depression) | .805 | .273 | -.175 | .92 | -.20 | .08 | -.070 | .74 | -.16 | .06 | -.06 | .25 | -.05 | .02 | -.02 | -.16 | .03 | -.01 | .01 |
| PP (Delusional Disorder) | .754 | .174 | .261 | .08 | .48 | .21 | -.060 | .06 | .36 | .16 | -.05 | .01 | .08 | .04 | -.01 | .02 | .13 | .06 | -.02 |
| Sum |  |  |  |  |  |  | | **6.51** | **3.76** | **-.75** | **-2.48** | **2.50** | **1.63** | **.86** | **-.98** | **-.93** | **.95** | **.15** | **.98** |

∑X^2^/ Sum of the squared loadings for our study: F1=11.22, F2 = 4.29, and F3 =1.79

∑Y^2^/ Sum of the squared loadings for Pignolo et al. (2017): F1 = 5.33, F2 = 3.05, F3= 1.87, and F4=1.54

We applied congruence coefficient (Φ) formula, obtaining a series of outcome inserted in Table 9.

**Appendix 5. Structure Matrix Results of Current Study and Dutch Study**

| **Scales** | **Arabic Study** | | | **Dutch Study** | | | | **Tucker’s Congruence Coefficients (TCC)** | | | | | | | | | | | |
| --- | --- | --- | --- | --- | --- | --- | --- | --- | --- | --- | --- | --- | --- | --- | --- | --- | --- | --- | --- |
|  | **Current Study** | | | (Rossi et al., 2007) | | | |  |  |  |  |  |  |  |  |  |  |  |  |
|  | F1IN | F2EX | F3PD | **F1** | **F2** | **F3** | **F4** | **F1*F1** | **F1*F2** | **F1*F3** | **F1*F4** | **F2*F1** | **F2*F2** | **F2*F3** | **F2*F4** | **F3*F1** | **F3*F2** | **F3*F3** | **F3*F4** |
| I (Schizoid) | .720 | .550 | -.285 | .65 | .24 | .41 | -.71 | .47 | .17 | .30 | -.51 | .36 | .13 | .23 | -.39 | -.19 | -.07 | -.12 | .20 |
| 2A (Avoidant) | .828 | .278 | -.194 | .78 | .15 | .43 | -.81 | .65 | .12 | .36 | -.67 | .22 | .04 | .12 | -.23 | -.15 | -.03 | -.08 | .16 |
| 2B (Depressive) | .865 | .391 | -.180 | .92 | .27 | .41 | -.61 | .80 | .23 | .35 | -.53 | .36 | .11 | .16 | -.24 | -.17 | -.05 | -.07 | .11 |
| 3 (Dependent) | .744 | .355 | -.026 | .81 | .20 | .34 | -.55 | .60 | .15 | .25 | -.41 | .29 | .07 | .12 | -.20 | -.02 | -.01 | -.01 | .01 |
| 4 (Histrionic) | -.319 | -.173 | .739 | -.54 | .00 | -.18 | .93 | .17 | .00 | .06 | -.30 | .09 | .00 | .03 | -.16 | -.40 | .00 | -.13 | .69 |
| 5 (Narcissistic) | .039 | -.001 | .802 | -.41 | .21 | .21 | .76 | -.02 | .01 | .01 | .03 | .00 | .00 | .00 | .00 | -.33 | .17 | .17 | .61 |
| 6A (Antisocial) | .618 | .877 | .166 | .21 | .99 | .35 | .10 | .13 | .61 | .22 | .06 | .18 | .87 | .31 | .09 | .03 | .16 | .06 | .02 |
| 6B (Sadistic) | .778 | .536 | .213 | .42 | .79 | .57 | .02 | .33 | .61 | .44 | .02 | .23 | .42 | .31 | .01 | .09 | .17 | .12 | .00 |
| 7 (Compulsive) | -.209 | -.763 | .288 | .30 | -.62 | -.07 | .09 | -.06 | .13 | .01 | -.02 | -.23 | .47 | .05 | -.07 | .09 | -.18 | -.02 | .03 |
| 8A (Negativistic) | .767 | .402 | .039 | .76 | .85 | .63 | -.31 | .58 | .65 | .48 | -.24 | .31 | .34 | .25 | -.12 | .03 | .03 | .02 | -.01 |
| 8B (Masochistic) | .829 | .587 | -.061 | .86 | .33 | .47 | -.61 | .71 | .27 | .39 | -.51 | .50 | .19 | .28 | -.36 | -.05 | -.02 | -.03 | .04 |
| S (Schizotypal) | .857 | .386 | -.045 | .82 | .32 | .67 | -.58 | .70 | .27 | .57 | -.50 | .32 | .12 | .26 | -.22 | -.04 | -.01 | -.03 | .03 |
| C (Borderline) | .868 | .590 | .010 | .87 | .56 | .45 | -.42 | .76 | .49 | .39 | -.37 | .51 | .33 | .27 | -.25 | .01 | .01 | .00 | .00 |
| P (Paranoid) | .777 | .046 | .213 | .55 | .31 | .93 | -.51 | .43 | .24 | .72 | -.40 | .03 | .01 | .04 | -.02 | .12 | .07 | .20 | -.11 |
| A (Anxiety) | .862 | .329 | .015 | .89 | .23 | .46 | .04 | .77 | .20 | .40 | .03 | .29 | .08 | .15 | .01 | .01 | .00 | .01 | .00 |
| H (Somatoform) | .798 | .348 | -.148 | .86 | .18 | .31 | -.51 | .69 | .14 | .25 | -.41 | .30 | .06 | .11 | -.18 | -.13 | -.03 | -.05 | .08 |
| N (Bipolar ):Manic | .801 | .476 | .079 | .56 | .56 | .59 | .04 | .45 | .45 | .47 | .03 | .27 | .27 | .28 | .02 | .04 | .04 | .05 | .00 |
| D (Dysthymia) | .870 | .428 | -.159 | .93 | .24 | .34 | -.61 | .81 | .21 | .30 | -.53 | .40 | .10 | .15 | -.26 | -.15 | -.04 | -.05 | .10 |
| B (Alcohol Dependence) | .634 | .825 | .065 | .33 | .67 | .35 | -.04 | .21 | .42 | .22 | -.03 | .27 | .55 | .29 | -.03 | .02 | .04 | .02 | .00 |
| T (Drug Dependence) | .513 | .848 | .140 | .01 | .69 | .20 | .20 | .01 | .35 | .10 | .10 | .01 | .59 | .17 | .17 | .00 | .10 | .03 | .03 |
| R (Posttraumatic Stress) | .852 | .341 | -.055 | .87 | .28 | .49 | -.46 | .74 | .24 | .42 | -.39 | .30 | .10 | .17 | -.16 | -.05 | -.02 | -.03 | .03 |
| SS (Thought Disorder) | .889 | .482 | -.092 | .93 | .33 | .47 | -.50 | .83 | .29 | .42 | -.44 | .45 | .16 | .23 | -.24 | -.09 | -.03 | -.04 | .05 |
| CC (Major Depression) | .857 | .409 | -.145 | .90 | .20 | .29 | -.54 | .77 | .17 | .25 | -.46 | .37 | .08 | .12 | -.22 | -.13 | -.03 | -.04 | .08 |
| PP (Delusional Disorder) | .759 | .265 | .287 | .45 | .34 | .85 | -.09 | .34 | .26 | .65 | -.07 | .12 | .09 | .23 | -.02 | .13 | .10 | .24 | -.03 |
| Sum |  |  |  |  |  |  | | **11.85** | **6.71** | **8.02** | **-6.49** | **5.93** | **5.19** | **4.30** | **-3.07** | **-1.31** | **.39** | **.22** | **2.09** |

∑X^2^/ Sum of the squared loadings for our study: F1=13.28, F2 = 5.96, and F3 =1.74

∑Y^2^/ Sum of the squared loadings for Rossi et al. (1998): F1 = 11.81, F2 = 5.29, F3= 5.50, and F4=5.98

We applied congruence coefficient (Φ) formula, obtaining a series of outcome inserted in Table 11.

**Appendix 6. Pattern Matrix Results of Current Study and Dutch Study**

| **Scales** | **Arabic Study** | | | **Dutch Study** | | | | **Tucker’s Congruence Coefficients (TCC)** | | | | | | | | | | | |
| --- | --- | --- | --- | --- | --- | --- | --- | --- | --- | --- | --- | --- | --- | --- | --- | --- | --- | --- | --- |
|  | **Current Study** | | | (Rossi et al., 2007) | | | |  |  |  |  |  |  |  |  |  |  |  |  |
|  | F1IN | F2EX | F3PD | **F1** | **F2** | **F3** | **F4** | **F1F1** | **F1F2** | **F1F3** | **F1F4** | **F2F1** | **F2F2** | **F2F3** | **F2F4** | **F3F1** | **F3F2** | **F3F3** | **F3F4** |
| I (Schizoid) | .607 | .436 | -.313 | .18 | .140 | .23 | -.59 | .11 | .08 | .14 | -.35 | .05 | .04 | .06 | -.17 | -.05 | -.04 | -.06 | .15 |
| 2A (Avoidant) | .812 | .135 | -.221 | .32 | .010 | .23 | -.65 | .28 | .01 | .20 | -.56 | -.03 | .00 | -.02 | .07 | -.06 | .00 | -.04 | .12 |
| 2B (Depressive) | .818 | .251 | -.210 | .79 | .020 | .04 | -.18 | .68 | .02 | .03 | -.15 | .01 | .00 | .00 | .00 | -.13 | .00 | -.01 | .03 |
| 3 (Dependent) | .701 | .246 | -.052 | .72 | -.010 | .02 | -.15 | .52 | -.01 | .01 | -.11 | .03 | .00 | .00 | -.01 | -.01 | .00 | .00 | .00 |
| 4 (Histrionic) | -.274 | -.066 | .749 | .03 | -.010 | -.11 | .94 | -.01 | .00 | .03 | -.29 | .00 | .00 | .00 | .00 | .02 | -.01 | -.08 | .69 |
| 5 (Narcissistic) | .066 | .060 | .799 | -.23 | .130 | .32 | .65 | -.01 | .01 | .01 | .03 | -.01 | .00 | .01 | .02 | -.19 | .10 | .26 | .52 |
| 6A (Antisocial) | .409 | .839 | .135 | -.14 | 1.020 | .06 | -.03 | -.04 | .30 | .02 | -.01 | -.11 | .78 | .05 | -.02 | -.03 | .22 | .01 | -.01 |
| 6B (Sadistic) | .690 | .453 | .183 | .15 | .600 | .31 | .09 | .10 | .40 | .21 | .06 | .04 | .16 | .08 | .02 | .04 | .14 | .07 | .02 |
| 7 (Compulsive) | .015 | -.751 | .305 | -.17 | -.651 | .23 | .06 | -.02 | -.09 | .03 | .01 | .14 | .53 | -.19 | -.05 | -.04 | -.16 | .06 | .01 |
| 8A (Negativistic) | .715 | .297 | .011 | .46 | .300 | .32 | -.13 | .33 | .22 | .23 | -.09 | .04 | .03 | .03 | -.01 | .03 | .02 | .02 | -.01 |
| 8B (Masochistic) | .722 | .475 | -.093 | .61 | .110 | .15 | -.28 | .43 | .08 | .11 | -.20 | .17 | .03 | .04 | -.08 | -.02 | .00 | .00 | .01 |
| S (Schizotypal) | .815 | .258 | -.076 | .45 | .050 | .43 | -.30 | .38 | .04 | .36 | -.25 | .01 | .00 | .01 | -.01 | -.01 | .00 | -.01 | .01 |
| C (Borderline) | .765 | .478 | -.024 | .76 | .330 | .02 | -.03 | .57 | .25 | .02 | -.02 | .21 | .09 | .01 | -.01 | .03 | .01 | .00 | .00 |
| P (Paranoid) | .840 | -.068 | .189 | .05 | -.010 | .90 | -.20 | .05 | -.01 | .83 | -.18 | -.02 | .00 | -.30 | .07 | .01 | .00 | .19 | -.04 |
| A (Anxiety) | .840 | .202 | -.015 | .90 | -.090 | .11 | .06 | .79 | -.08 | .10 | .05 | -.04 | .00 | .00 | .00 | .02 | .00 | .00 | .00 |
| H (Somatoform) | .759 | .219 | -.176 | .90 | -.080 | -.05 | -.03 | .71 | -.06 | -.04 | -.02 | .00 | .00 | .00 | .00 | -.12 | .01 | .01 | .00 |
| N (Bipolar ):Manic | .730 | .374 | .049 | .54 | .280 | .28 | .35 | .39 | .20 | .20 | .26 | .09 | .05 | .05 | .06 | .06 | .03 | .03 | .04 |
| D (Dysthymia) | .812 | .291 | -.190 | .88 | -.010 | -.17 | -.15 | .74 | -.01 | -.14 | -.13 | .05 | .00 | -.01 | -.01 | -.12 | .00 | .02 | .02 |
| B (Alcohol Dependence) | .439 | .773 | .034 | .09 | .610 | .10 | -.03 | .03 | .21 | .03 | -.01 | .06 | .42 | .07 | -.02 | .01 | .07 | .01 | .00 |
| T (Drug Dependence) | .303 | .824 | .112 | -.09 | .730 | .03 | .06 | -.02 | .13 | .01 | .01 | -.07 | .57 | .02 | .05 | -.02 | .14 | .01 | .01 |
| R (Posttraumatic Stress) | .823 | .210 | -.085 | .81 | -.020 | .15 | .00 | .70 | -.02 | .13 | .00 | -.02 | .00 | .00 | .00 | -.03 | .00 | -.01 | .00 |
| SS (Thought Disorder) | .818 | .351 | -.125 | .88 | .040 | .07 | -.51 | .73 | .03 | .06 | -.43 | .11 | .00 | .01 | -.06 | -.06 | .00 | .00 | .04 |
| CC (Major Depression) | .805 | .273 | -.175 | .95 | -.050 | -.12 | -.03 | .79 | -.04 | -.10 | -.03 | .04 | .00 | -.01 | .00 | -.12 | .01 | .02 | .00 |
| PP (Delusional Disorder) | .754 | .174 | .261 | .12 | .030 | .79 | .04 | .09 | .02 | .62 | .03 | -.01 | .00 | -.04 | .00 | .04 | .01 | .23 | .01 |
| Sum |  |  |  |  |  |  | | **8.34** | **1.70** | **3.10** | **-2.40** | **.77** | **2.70** | **-.13** | **-.17** | **-.77** | **.55** | **.72** | **1.64** |

∑X^2^/ Sum of the squared loadings for our study: F1=11.22, F2 = 4.29, and F3 =1.79

∑Y^2^/ Sum of the squared loadings for Rossi et al. (1998): F1 = 7.82, F2 = 3.08, F3= 2.29, and F4=2.79

We applied congruence coefficient (Φ) formula, obtaining a series of outcome inserted in Table 12.

**Vita**


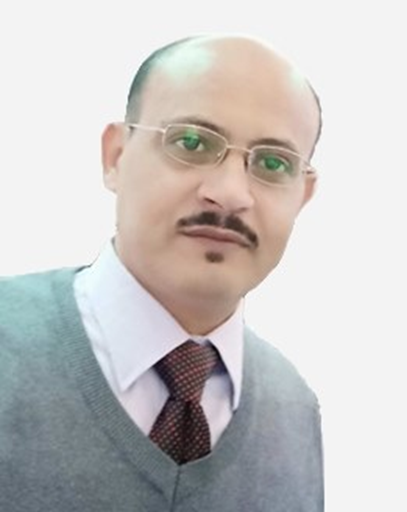


Dr. Nasser Alareqe

He obtained his PhD in Educational Psychology and Counselling from International Islamic University Malaysia (IIUM). He has various experiences conducting workshops (over 200 workshops) in areas of Measurements and Advanced Statistics for postgraduate researchers and Staff of Malaysian Universities using SPSS, SAS, R Project, Stata, AMOS, EQS, LISREL, HLM, Mplus, and SmartPLS. He also did workshops in Systematic Review and Meta-Analysis, Rasch Analysis-Winsteps and EQS-Item Response Theory (IRT). He is a lecturer of qualitative and mixed methods in Malaysia. He is a reviewer for PLOS ONE, SAGE and Journal of Medical Imaging and Health Informatics. E-mail: [nalareqe@yahoo.com.](mailto:nalareqe@yahoo.com.com)


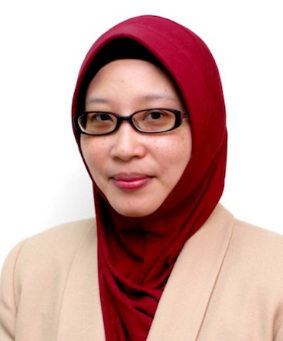


Prof. Dr. Samsilah Roslan

Prof. Dr. Roslan is a full professor in Educational Psychology at University Putra Malaysia (UPM). She is dean of Faculty of Educational studies at UPM. She was involved in 36 research and 9 consultancy projects with various bodies and agencies in Malaysia. Prof. Dr. Roslan published more than one hundreds articles in different international Journals. She is consultant editor for local and international journals. She was hold several local and international awards. Prof. Dr. Roslan spoke three languages: Malay, Arabic and English.


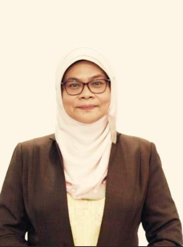


**Associate Professor Dr. Nor Aniza Ahmad**

Dr. Nor Aniza Ahmad is an Associate Professor at the Universiti Putra Malaysia (UPM), in the Faculty of Educational Studies. Her expertise is on the educational psychology and learning diversity. She is interested in understanding learning sciences research and shaping learning in a number of settings such as schools, workplaces, and higher education. She is actively involved in academic research and has becomes a lead researcher and co-researcher in 14 research grants from UPM, Ministry of Education and international collaboration. Her research interests include psychology of learning, gender study, student’s diversity and motivation. She also actively contributes her expertise to the community and industry program. Dr. Nor Aniza Ahmad is a reviewer and member of an editorial board for national and international journal. She has been also a competent supervisor for many PhD students and Post-Doctoral Researchers at UPM. E-mail: [nor_aniza@upm.edu.my](mailto:nor_aniza@upm.edu.my).

Prof. Dr. Mohamad Sahari Nordin is one of the established consultants in Applied Statistics in Malaysia. He is consultant for Ministry of Education in Malaysia and Arabic Gulf. He graduated from Florida (USA). He has been teaching Psychological Courses, Research Methods, Advanced Statistics, and SEM-AMOS with EQS courses for PhD level at IIUM since 1998. He has been also a supervisor for many PhD students and Post-Doctoral Researchers at IIUM. E-mail: [sahariuiam@gmail.com](mailto:sahariuiam@gmail.com). <http://www.iium.edu.my/staff-details?id=6516>


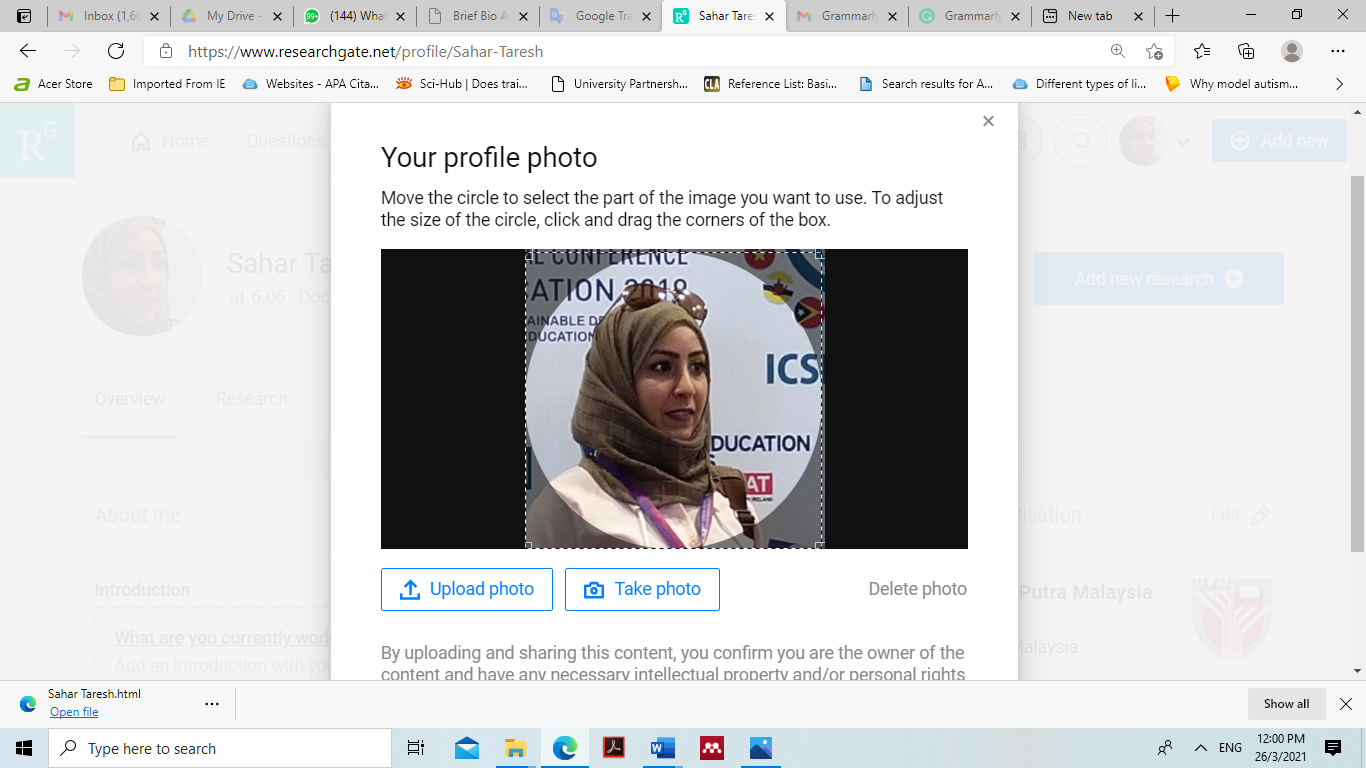


Sahar Mohammed Taresh is a lecturer in the Kindergarten department, Taiz University, Yemen. She obtained her master's degree at Taiz university. Now she is a Ph.D. candidate in Universiti Putra Malaysia (UPM). She has publications in childhood and adolescence. She obtained some medals in innovation and competition and education. Now, she is a reviewer for SAGE and the Journal of international educational studies. <https://www.researchgate.net/profile/Sahar-Taresh?ev=hdr_xprf>.
